# Mathematical kinetic modelling followed by in vitro and in vivo assays reveal the bifunctional rice GTPCHII/DHBPS enzymes and demonstrate the key roles of OsRibA proteins in the vitamin B2 pathway

**DOI:** 10.1186/s12870-024-04878-z

**Published:** 2024-03-26

**Authors:** Maria Faustino, Tiago Lourenço, Simon Strobbe, Da Cao, André Fonseca, Isabel Rocha, Dominique Van Der Straeten, M. Margarida Oliveira

**Affiliations:** 1 Laboratory of Plant Functional Genomics, Instituto de Tecnologia Química E Biológica António Xavier, Universidade Nova de Lisboa, Oeiras, 2780-157 Portugal; 2 Laboratory of Functional Plant Biology, Department of Biology, Ghent University, K. L. Ledeganckstraat 35, Gent, B-9000 Belgium; 3 Laboratory of Systems and Synthetic Biology, Instituto de Tecnologia Química E Biológica António Xavier, Universidade Nova de Lisboa, Oeiras, 2780-157 Portugal; 4 Present Address: University of Geneva, Quai E. Ansermet 30, Geneva, 1211 Switzerland

**Keywords:** Biofortification, 2,5-diamin-6-ribosylamino-4(3H)-pyrimidinone-5-phosphate, 3,4-dihydroxy-2-butanone-4-phosphate, Limiting step, *Oryza sativa*, RibA

## Abstract

**Background:**

Riboflavin is the precursor of several cofactors essential for normal physical and cognitive development, but only plants and some microorganisms can produce it. Humans thus rely on their dietary intake, which at a global level is mainly constituted by cereals (> 50%). Understanding the riboflavin biosynthesis players is key for advancing our knowledge on this essential pathway and can hold promise for biofortification strategies in major crop species. In some bacteria and in Arabidopsis, it is known that RibA1 is a bifunctional protein with distinct GTP cyclohydrolase II (GTPCHII) and 3,4-dihydroxy-2-butanone-4-phosphate synthase (DHBPS) domains. Arabidopsis harbors three RibA isoforms, but only one retained its bifunctionality. In rice, however, the identification and characterization of RibA has not yet been described.

**Results:**

Through mathematical kinetic modeling, we identified RibA as the rate-limiting step of riboflavin pathway and by bioinformatic analysis we confirmed that rice RibA proteins carry both domains, DHBPS and GTPCHII. Phylogenetic analysis revealed that OsRibA isoforms 1 and 2 are similar to Arabidopsis bifunctional RibA1. Heterologous expression of *OsRibA1* completely restored the growth of the *rib3∆* yeast mutant, lacking DHBPS expression, while causing a 60% growth improvement of the *rib1∆* mutant, lacking GTPCHII activity. Regarding *OsRibA2*, its heterologous expression fully complemented GTPCHII activity, and improved *rib3∆* growth by 30%. In vitro activity assays confirmed that both OsRibA1 and OsRibA2 proteins carry GTPCHII/DHBPS activities, but that OsRibA1 has higher DHBPS activity. The overexpression of *OsRibA1* in rice callus resulted in a 28% increase in riboflavin content.

**Conclusions:**

Our study elucidates the critical role of RibA in rice riboflavin biosynthesis pathway, establishing it as the rate-limiting step in the pathway. By identifying and characterizing *OsRibA1* and *OsRibA2*, showcasing their GTPCHII and DHBPS activities, we have advanced the understanding of riboflavin biosynthesis in this staple crop. We further demonstrated that *OsRibA1* overexpression in rice callus increases its riboflavin content, providing supporting information for bioengineering efforts.

**Supplementary Information:**

The online version contains supplementary material available at 10.1186/s12870-024-04878-z.

## Background

Riboflavin (vitamin B_2_) is the precursor of the essential cofactors, flavin adenine dinucleotide (FAD) and flavin mononucleotide (FMN), which are implicated in many metabolic processes [1, 2]. These flavoenzymes are extraordinarily versatile at the chemical level, having the ability to catalyze both redox and non-redox processes [3] and being of paramount importance for multiple biological activities [4]. To name a few, flavoenzymes are involved in primary energy metabolism [5], synthesis of key cell constituents [6] and secondary metabolites [7], synthesis and degradation of neurotransmitters and coenzymes [8], DNA repair [9], gene expression regulation [10] and control of circadian clock [11].

In contrast to plants, fungi and bacteria, humans do not have the ability to produce riboflavin de novo and rely solely on dietary intake to meet the daily requirements [2, 5, 12]. The deficiency in riboflavin, known as ariboflavinosis, is a global health concern that can result in various disorders, such as cardiovascular diseases, anemia, cancer and neurological and developmental conditions [13]. The daily average recommended vit. B2 intake set by the European Food Safety Authority (EFSA) [14] has not yet been met by a large part of the population [14]. Although more prevalent in developing countries [13, 15–18], riboflavin deficiency affects 50% of the population [19], it is also common in developed countries [20], especially in vulnerable groups such as pregnant and lactating women, as well as in infants and elderly people [21, 22]. Monotonous, cereal-based diets are a key contributing factor to riboflavin deficiency in developing countries [23, 24], highlighting the need for new strategies to increase the quality of their diets and their nutritional intake.

Cereal processing, although crucial in improving the shelf life of cereals, such as rice [25–27], results in a significant loss of riboflavin, which is mainly found in the pericarp [28]. Only in rice, the milling process decreases riboflavin content by 38% [29]. Thus, the use of genetically improved cereals, enriched in riboflavin at the endosperm level, can be a feasible and cost-effective strategy to address riboflavin deficiency in populations with limited access to diverse diets and good health facilities [30, 31].

The biosynthetic pathway of riboflavin is a highly conserved process originating from two precursors, guanine triphosphate (GTP) and ribulose 5-phosphate (Rub5P) [32]. Minor evolutionary differences exist in different lineages, such as the order of intermediary steps and the fusion of riboflavin biosynthesis proteins acting as bifunctional enzymes (RIB) [32, 33]. For example, the reduction/deamination reactions of the riboflavin pyrimidine precursor occur in the same sequence in fungi and archaea, where reduction is followed by deamination, while in eubacteria and plants the sequence is reversed, with deamination preceding reduction [32, 34]. Another example is the bifunctional enzyme RibA, which bears both GTP cyclohydrolase II (GTPCHII) and dihydroxybutanone phosphate synthase (DHBPS) activities and is utilized by plants and the Gram-positive bacterium *Bacillus subtilis* to catalyze the formation of the two precursors [35, 36]. In contrast, *Escherichia coli* and yeast use two separate monofunctional enzymes (ribA and ribB) [37]. The fused enzyme has been suggested to have a kinetic advantage to consume the two substrates stoichiometrically [38]. Although riboflavin biosynthesis and metabolism are well understood, especially in microorganisms [27, 39], there is still a lack of clear genetic support for the function of most plant riboflavin synthesis enzymes [40].

**Fig. 1:**
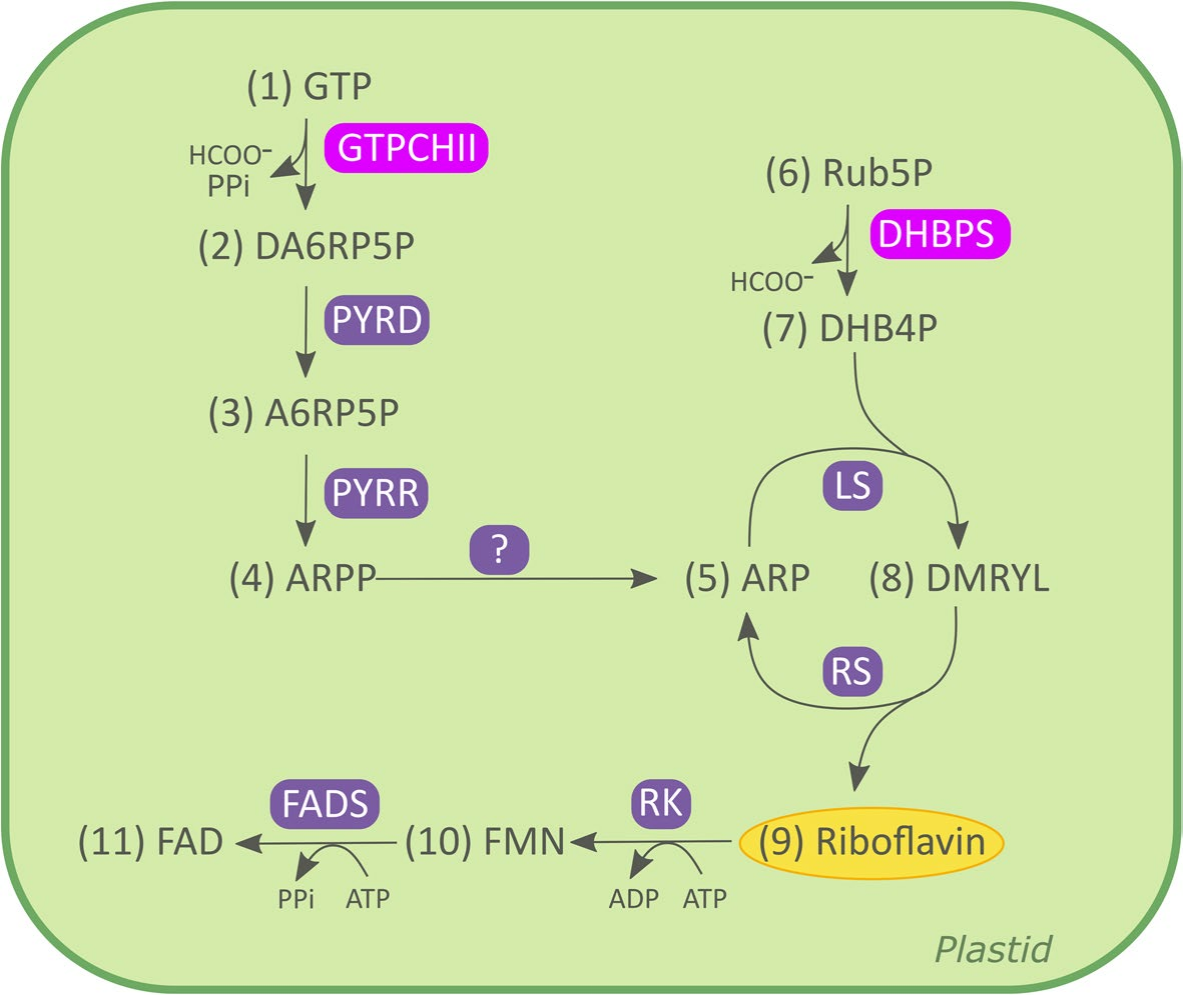
Riboflavin biosynthetic pathway. The riboflavin pathway starts with the hydrolytic release of formate and inorganic pyrophosphate from (1) GTP catalyzed by GTP cyclohydrolase II (GTPCHII) giving rise to the pyrimidine derivative: (2) 2,5-diamin-6-ribosylamino-4(3*H*)-pyrimidinone 5’-phosphate [32] (DA6RP5P). This product is converted into (4) 5-amino-6-ribitylamino-2,4(1H,3H)-pyrimidinedione 5’-phosphate (ARPP) via (3) 5-amino-6-ribosylamino-2,4(1*H*, 3*H*)-pyrimidinedione 5'-phosphate (A6RP5P) reactions catalyzed by 2,5-diamino-6-hydroxy-4-(5-phosphoribosylamino)pyrimidine deaminase (PYRD) and 5-amino-6-(5-phosphoribosylamino)uracil reductase (PYRR). ARPP is converted into ARP by a hypothetical phosphatase, not yet identified in plants [39]. The resulting pyrimidine derivative yields (8) 6,7-dimethyl-8-ribityllumazine (DMRYL) by condensation with (7) 3,4-dihydroxy-2-butanone 4-phosphate (DHB4P) catalyzed by lumazine synthase, (LS) which is obtained from (6) ribulose 5-phosphate (Rub5P) by a skeletal rearrangement (by the action of 3,4-dihydroxy-2-butanone 4-phosphate synthase (DHBPS)) [39]. The final step of the biosynthetic pathway yields (9) riboflavin and the pyrimidine catalyzed by riboflavin synthase (RS). Adapted from Fischer and Bacher (2006) [39]. Abbreviations: (1) GTP, guanosine-5'-triphosphate; (2) 2,5-diamin-6-ribosylamino-4(3H)-pyrimidinone 5’-phosphate (DA6RP5P); (3) 5-amino-6-ribosylamino-2,4(1H, 3H)-pyrimidinedione 5'-phosphate (A6RP5P); (4) 5-amino-6-ribitylamino-2,4(1H,3H)-pyrimidinedione 5’-phosphate (ARPP); (5) 5-amino-6-ribitylamino-2,4 (1H,3H)-pyrimidinedione (ARP); (6) ribulose 5-phosphate (Rub5P); (7) 3,4-dihydroxy-2-butanone 4-phosphate (DHB4P); (8) 6,7-dimethyl-8-ribityllumazine (DMRYL); (9) riboflavin; (10) flavin mononucleotide (FMN); (12) flavin dinucleotide (FAD). HCOO−, formate; PPi, pyrophosphate; Bifunctional RibA, GTP cyclohydrolase II/3,4-dihydroxy-2-butanone 4-phosphate synthase; PYRD, 2,5-diamino-6-hydroxy-4-(5-phosphoribosylamino)pyrimidine deaminase; PYRR, 5-amino-6-(5-phosphoribosylamino)uracil reductase; LS, lumazine synthase; RS, riboflavin synthase; RK, riboflavin kinase; FADS, FAD synthetase

The pathway for de novo synthesis is illustrated in Fig. 1. Briefly, riboflavin building blocks are guanine triphosphate (GTP) and ribulose 5-phosphate (Rub5P) [32]. In plants, the formation of 2,5-diamin-6-ribosylamino-4(3H)-pyrimidinone 5-phosphate (DA6RP5P) from GTP and of 3,4-dihydroxy-2-butanone 4- phosphate (DHB4P) from Rub5P is catalyzed by GTP cyclohydrolase II (GTPCHII) and 3,4-dihydroxy-2-butanone 4-phosphate synthase (DHBPS), respectively [41]. In Arabidopsis, these two steps can be performed either by the bifunctional enzyme RibA1 or by the consecutive action of the monofunctional GTPCHII (RibA2) and DHBPS (RibA3) [35]. The formation of DA6RP5P is followed by its deamination, reduction, and dephosphorylation into 5-amino-6-ribityl-aminouracil (ARPP). Subsequently, ARP and DHB4P are condensed to form 6,7-dimethyl-8-ribityl lumazine (DMRYL) which is finally converted into riboflavin [41].

Biofortification of riboflavin in rice has been previously performed by overexpressing six out of the seven genes that constitute the yeast biosynthetic pathway [12]. However, recent trends in biofortification suggest a shift towards the overexpression of endogenous genes, reflecting public consumer preferences [42, 43]. In addition, a reliable understanding of endogenous riboflavin metabolism allows for more precise metabolic engineering and thus further opportunities for engineering approaches. In this study, we focused on better understanding the rice riboflavin pathway by identifying its rate limiting enzyme. Through kinetic modeling, we revealed the bifunctional GTPCHII/DHBPS, RibA, as the bottleneck of the pathway, a gene not yet identified and characterized in rice. By exploring in silico data and performing heterologous expression and in vitro activity assays, we could identify the gene as LOC_Os08g37605 (OsRibA1). In addition, i*n vivo* overexpression studies in rice callus supported its bifunctional activity as GTPCHII/DHBPS, and resulted in a significant increase in riboflavin content, suggesting OsRibA1 as an interesting candidate for biofortification studies.

## Materials and methods

### Kinetic model

To construct the model, we first established the topology of the riboflavin biosynthesis pathway network based on the knowledge on the metabolism of riboflavin production in plants [39–41] and incorporated kinetic constants obtained from the literature [32, 44–51]. Using this information, we developed a model assuming a simple Michaelis–Menten type reaction for enzyme kinetics in the pathway:

$$v=\frac{{V}_{max}\times [S]}{{K}_{m}+[S]}$$

Where* v* is the velocity of the enzyme that catalyzed the reaction, S is the substrate concentration in (nM), V_max_ is the maximum reaction velocity (nmol/mg.min^−1^), and K_m_ (nM) is the substrate concentration giving rise 1/2 V_max_. However, when required (LS and RK reactions), the two-substrate kinetic equation was modeled using the following equation:

$$\frac{{V}_{max}\times \left[A\right]\times [B]}{{K}_{mA} \times {K}_{mB}+\left[A\right]\times {K}_{mB}+\left[B\right]\times {K}_{mA}+\left[A\right]\times [B]}$$

A is the concentration of one of the substrates, and B is the concentration of the other substrate, both in nM.

A set of ordinary differential equations was constructed using the previously mentioned expressions to account for the time dependence of the metabolite concentration. The initial metabolite concentrations were estimated based on previously published studies [52–54]. Time course simulations were conducted to evaluate riboflavin production over time, employing the deterministic model (LSODA) from COPASI [55]. The simulation was run for a duration of 1500s to ensure the formation of a plateau. Details on all reactions and respective stoichiometry are shown in Table S1, while the kinetic parameters are outlined in Table S2.

Kinetic equations and their respective parameters were obtained through manual curation of an extensive survey of scientific literature [32, 34, 44–51] and/or public databases such as BRENDA [56], KEGG [57], BioCyc [58] and UniProt [59]. In addition, genomic data, obtained from MSU Rice Genome Annotation Project [60] and National Center for Biotechnology Information (NCBI) [61], was also integrated into the model. For reactions where kinetic parameters were not available in the literature (import of GTP, import of R5P, FADS, and export of FAD reactions), the stoichiometric model of vitamin B2 metabolism (Figure S1, Tables S3 and S4) was used to predict the reaction flux.

### Identification and phylogenetic analysis of *OsRibA*

BLASTt algorithm was employed to identify homologs of Arabidopsis RibA1 (AT5G64300) in *Oryza sativa* japonica cv. Nipponbare genome. Alignments were made with MUSCLE [62] and visualized in Jalview [63]. The expression profile of *OsRibA1*, *OsRibA2* and Arabidopsis *RibA1* were compared at different stages of plant development through the analysis of the publicly available data at Genevestigator (NEBION) [64].

To determine the evolutionary relationships among RibA1 orthologs, we generated a phylogenetic dendrogram using the maximum likelihood method and evaluated the tree with 1000 bootstrap replicates using MEGAX [65, 66]. The final dendrograms of the bootstrap consensus were displayed using FigTree v1.4.2. For the identification of *AtRibA1* orthologs, the full-length Arabidopsis RibA1 was used as a query to run Blastp. Pfam [67] and ScanProsite [68] were used to evaluate the presence of GTPCHII and DHBPS domains in the putative ortholog proteins. Smart Model Select (SMS) [69] was used to select WAG model [70].

### Gene cloning and vector construction

The predicted DNA sequences of *OsRibA1* and *OsRibA2* were obtained from MSU Rice Genome Annotation Project [71] and the coding sequences were amplified from *Oryza sativa* japonica cv. Nipponbare cDNA (Table S5). The obtained DNA fragments were cloned into pJET 1.2 vector (Thermo Scientific CloneJET PCR Cloning Kit, Waltham, Massachusetts, EUA). Gibson assembly (Gibson assembly master mix, Neb, Ipswich, Massachusetts, EUA) was used to generate pPGK-*OsRibA1* and pPGK-*OsRibA2* (Figure S2), under the control of 3-phosphoglycerate kinase (PGK) promoter, one of the most efficient yeast promoters for constitutive expression [72], and employed in yeast complementation studies. The sequences were verified by DNA sequencing. *E. coli* DH5α strain was grown in LB medium (supplemented with the appropriate antibiotic) and used for bacterial transformation. For rice callus transformation, binary vectors were generated by cloning *OsRibA1*, *OsRibA2*, and *AtRibA1* (positive control) into Gateway™ pDONR™221 vector (Thermo Scientific, Waltham, Massachusetts, EUA) and recombined with the binary vector pH7m24GW,3 using Gateway® LR Clonase™ Enzyme Mix kit (Thermo Fisher Scientific) (Figure S2), according to the manufacturer’s guidelines. *OsRibA1* and *OsRibA2* were amplified from pJET, while *AtRibA1* was amplified from *Arabidopsis* cDNA (Table S5). As negative control, a point mutation generating an early stop codon was introduced into *OsRibA1* and *OsRibA2* using QuickChange – Site directed mutagenesis kit (Agilent, Santa Clara, California, EUA). For the in vitro activity assays, pDONR™221-*OsRibA1*, and pDONR™221- *OsRibA2* were recombined with a plasmid carrying a maltose binding protein. All the primers used in this study are listed in Table S5.

### Yeast strains and culture conditions

Yeast strains used in this study included: BY4741 (MATa; his3Δ1; leu2Δ0; met15Δ0; ura3Δ0), Y03059 (BY4741; MATa; his3Δ1; leu2Δ0; met15Δ0; ura3Δ0; YBL033c::kanMX4) and Y24321 (BY4743; MATa/MATα; his3Δ1/his3Δ1; leu2Δ0/leu2Δ0; LYS2/lys2Δ0; met15Δ0/MET15; ura3Δ0/ura3Δ0; YDR487c/YDR487c::kanMX4), obtained from EUROSCARF, Germany. Yeast strains were grown in YPD medium (10 g/L yeast extract, 20 g/L peptone, 20 g/L D-glucose) and synthetic medium (20 g/L glucose, 6.7 g/L YNB and 19 g/L agar, arginine 20 mg/L, Isoleucine 30 mg/L, lysine 30 mg/L, methionine 20 mg/L, phenylalanine 50 mg/L, threonine 200 mg/L, tyrosine 200 mg/L, valine 150 mg/L, adenine 100 mg/L, leucine 100 mg/L) with specific requirements (SD).

### Yeast complementation assays

Lithium-Acetate (LiAc) method [73] was used to transform all yeast strains. The mutants transformed with the pPGK empty vector were used as negative control while the wild type transformed with the empty pPGK was used as positive control. For phenotypic growth assays, early exponential phase cultures (OD_600nm_ = 0.4) were sequentially diluted (1/10, 1/100, 1/1000, and 1/10000) and spotted (5μL) in SD medium (lacking uracil) containing 0 mg/mL, 50 mg/mL or 100 mg/mL of riboflavin (Sigma, St. Louis, Missouri, EUA). The plates were incubated for 3 days at 30 °C, after which the colonies were photographed. For the growth curves, growth was monitored by optical density (OD_600nm_) measurements using the TECAN Infinite 200 Pro plate reader. 24-well plates (CELLSTAR, Greiner Bio-One) were inoculated with 500 µL (OD_600nm_ = 0.05) and incubated at 30 °C for 36 h (200 rpm).

### Plant material and transformation

Following established methods [74], the above-described binary vectors were introduced in *Agrobacterium tumefaciens* strain EHA105. Embryogenic rice calluses were obtained from *Oryza sativa* L. spp japonica cv. Nipponbare. Seeds were obtained from plants grown in glasshouse from April to September 2020 at ITQB NOVA (latitude: 38° 41′ 38" (38.694 N), longitude: 9° 19′ 7" (-9.318 W)), under natural photoperiod. Seeds produced from this plants were used to induce embryogenic calluses following an established protocol [75]. Embryogenic calluses were transformed by *Agrobacterium tumefaciens* strain EHA105 as described by Upadhyaya et al*.* (2000) [75] and transformants were selected with 1µl/mL hygromycin. Aiming to develop highly embryogenic tissues with a large number of somatic embryos, calluses were maintained in embryogenic induction medium (EIM) [76] for one month. The presence of the *hpt*II gene was verified using PCR amplification (Table S5). Twelve positive independent lines for each transgene were randomly pooled, freeze-dried (FreeZone Plus 4.5 Liter Cascade, LABCONGO), and stored at -80 °C.

### Riboflavin extraction and quantification

The homogenized rice calluses were extracted with 1 mL of 50 mM of phosphate buffer containing riboflavin-^13^C,^15^N_2_ as internal standard. The extract was vortexed for 10 s and stored for 2 h at 4 ℃. Then, it was purified with Amicon 3KDa centrifugal filter (15,900 rcf at 4 °C for 20 min) before loading to liquid chromatography coupled with tandem mass spectrometry system (LC–MS/MS). The LC–MS/MS was a Waters ACQUITY UPLC and an Applied Biosystems API 4000 MS equipped with an electrospray ionization source. The extracted riboflavin was separated on a Waters ACQUITY UPLC HSS T3 Column (2.1 mm X 150 mm, 100Å, 1.8 µm) equipped with a Waters ACQUITY UPLC HSS T3 VanGuard Pre-column (2.1 mm X 5 mm, 100Å, 1.8 µm) maintained at 45 ℃.

### Protein overexpression and isolation

The plasmids pMAL-*OsRibA1*, pMAL-*OsRibA2*, and the empty vector (used as negative control) were transformed into *E. coli* Rosetta (DE3) pLysS competent cells. The cells were grown in LB medium containing ampicillin (100μg/mL) and chloramphenicol (30μg/mL) at 37°C until reaching an OD_600nm_ of 0.6. Protein expression was induced with 100 μM of isopropyl b-D-thiogalactopyranoside (IPTG) and grown for 6 h at 28°C.

Cells were harvested by centrifugation (3500 g, 20 min, 4°C) and resuspended into 20 mM sodium phosphate (pH 7.5), 500 mM NaCl, 10 mM imidazole, 250 μM MgCl_2_, 1.25 mM PMSF, 1 × complete protease inhibitor (Roche) and 8 μg/mL DNase. Enzymatic lysis was performed by adding lysozyme (50 mg/mL), and cells were incubated at 4°C for 1 with agitation. The soluble fraction was obtained by centrifugation for 1h at 18, 000 × g at 4°C and filtered through a 0.45 μm filter before injection on a MBP-trap HP column (Sigma-Aldrich, Missouri, EUA). *Os*RibA1 and *OsRibA2* were eluted with 10 mM maltose, 20 mM Tris–HCl pH 7.4, 200 mM NaCl, and 1 mM EDTA (ethylene diamine tetra-acetic acid). Protein purity was assessed by sodium dodecyl sulphate polyacrylamide gel electrophoresis analysis.

### In vitro activity assays

The enzymatic assays were performed according to Bacher and colleagues (1997) [77], with minor modifications. GTP cyclohydrolase II (GTPCHII) activity was assayed in 100 μL reaction mixtures, containing 100 mM Tris–HCl pH 7.8, 5 mM MgCl_2_, 5 mM DTT, 100 μM GTP, 5mM diacetyl using either *OsRibA1*, *OsRibA2* (0.5, 0.2, 01 μg/μL) or free MBP (0.1 μg/μL) produced and purified as described above. GTPCHII product (2,5-diamin-6-ribosylamino-4(3H)-pyrimidinone 5’-phosphate) reacts with diacetyl yielding 6,7-dimethylpterin (ʎ = 400nm), which was the compound detected. For DHBPS activity, assay mixtures were prepared in 100 μL containing 100 mM Tris–HCl pH 7.8, 5 mM MgCl_2_, 5 mM DTT, 100 μM D-ribulose 5-phosphate, 5-diamino-6-ribitylamino-2,4(1H,3H) pyrimidinedione, lumazine synthase of *B. subtilis* (0.5 U/mL) and either *OsRibA*1*, OsRibA2* (0.2, 0.1, 0.05 μg/μL) or free MBP (0.1 μg/μL). 6,7-dimethyl-8- ribityllumazine, the product of lumazine synthase was monitored (λ = 490 nm). Reaction mixtures were incubated at 37°C for 1 h in Infinite 200 PRO multimode plate reader (Tecan). The purified *OsRibA1* and *OsRibA2* proteins were used to assess their respective activities in these assays and all reactions were performed in triplicate.

### Data analysis

The growth rate was used to compare the growth of the yeast mutants and the wild type. Specifically, growth curve data was transformed into their natural logarithm, and the exponential phase was selected for analysis. A non-linear regression was applied, and the slope of the resulting curve was used to compare the different groups using One-way ANOVA. To compare the concentration of riboflavin in different callus lines, One-way ANOVA was used. Significant differences in means are indicated for datasets in which P-values below 0.05 are considered significant, and p-values below 0.01 are considered very significant. The statistical analysis was performed using GraphPad8.

## Results

### Bifunctional RibA is the rate-limiting enzyme of riboflavin biosynthesis in plants

**Fig. 2:**
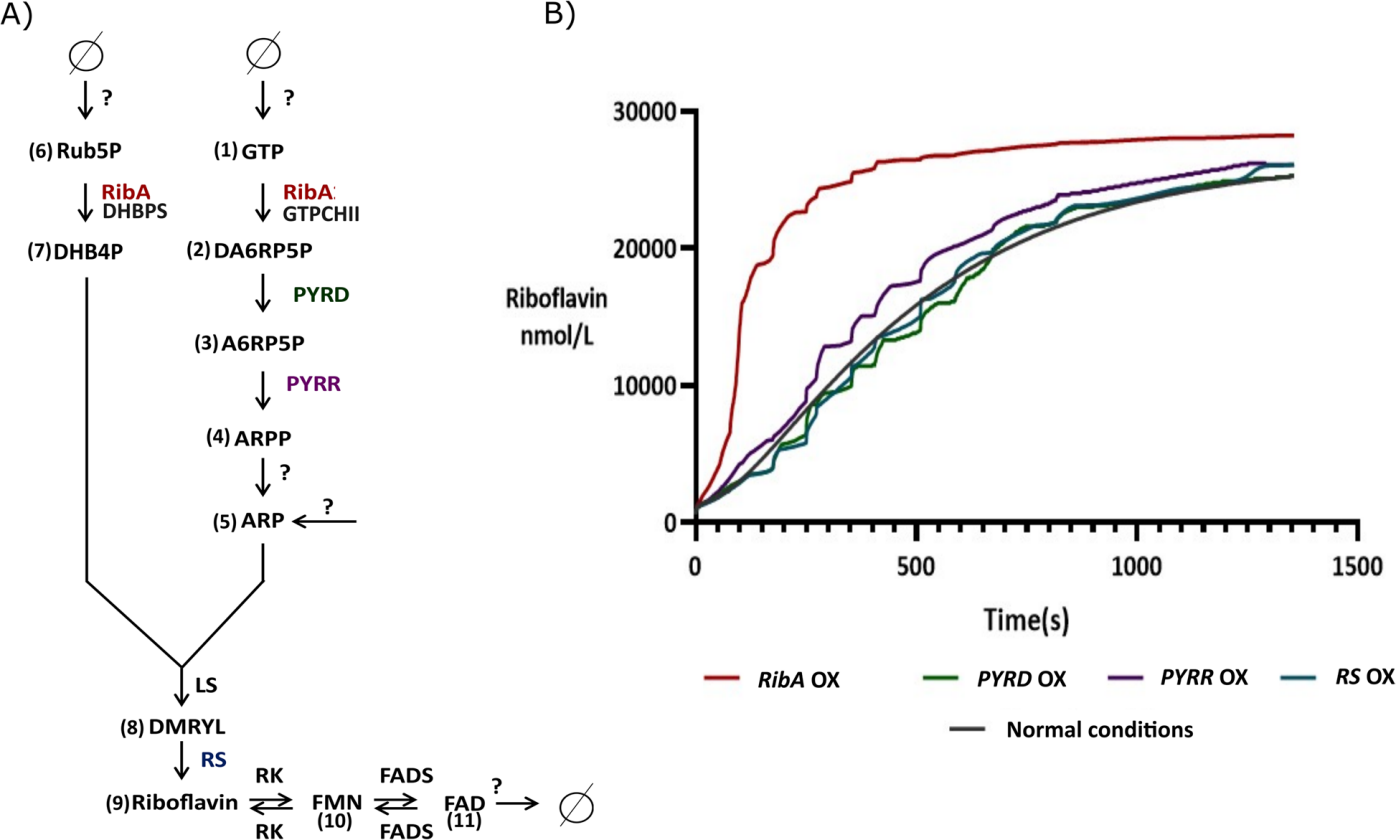
Riboflavin biosynthesis pathway and kinetic model simulation results. **A** Simplified riboflavin biosynthesis. The bifunctional RibA (GTP cyclohydrolase II/ 3,4-dihydroxy-2-butanone 4-phosphate synthase) mediates the synthesis of (7) DHB4P and (2) DA6RP5P which is converted into (5) ARP by the serial action of PYRD, PYRR and a hypothetical phosphatase not yet identified in plants [39]. LS condensates (7) DHBPS and (5) ARP to yield (8) DMRYL, which is converted into (9) riboflavin by the action of RS [33]. The translocation of ARP into the plastid and the translocation of FAD into the cytosol are hypothesized, although no corresponding transporters have been identified in plants thus far [39]. **B** Simulation results for riboflavin production along time in the non-optimized model (Green, Normal conditions), overexpressing PYRD (pink), overexpressing PYRR (purple), overexpressing RS (gray) and overexpressing bifunctional RibA (dark red). Abbreviations: (1) GTP, guanosine-5'-triphosphate; (2) DA6RP5P, 2,5-diamin-6-ribosylamino-4(3H)-pyrimidinone 5’-phosphate; (3) A6RP5P, 5-amino-6-ribosylamino-2,4(1H, 3H)-pyrimidinedione 5'-phosphate; (4) ARPP, 5-amino-6-ribitylamino-2,4(1H,3H)-pyrimidinedione 5’-phosphate; (5) ARP, 5-amino-6-ribitylamino-2,4 (1H,3H)-pyrimidinedione (ARP); (6) Rub5P, ribulose 5-phosphate; (7) DHB4P, 3,4-dihydroxy-2-butanone 4-phosphate; (8) DMRYL, 6,7-dimethyl-8-ribityllumazine; (9) riboflavin; (10) FMN, flavin mononucleotide; (12) FAD, flavin dinucleotide. Bifunctional RibA, GTP cyclohydrolase II/3,4-dihydroxy-2-butanone 4-phosphate synthase; PYRD, 2,5-diamino-6-hydroxy-4-(5-phosphoribosylamino)pyrimidine deaminase; PYRR, 5-amino-6-(5-phosphoribosylamino)uracil reductase; LS, lumazine synthase; RS, riboflavin synthase; RK, riboflavin kinase; FADS, FAD synthetase

Aiming to identify the bottleneck of the riboflavin pathway in plants, a kinetic model was assembled, based on the riboflavin metabolic pathway, comprising 12 metabolites and 14 reactions, including external transport (Table S1, Figure S3). To evaluate the dynamics of riboflavin production over time, we performed a time-course simulation using COPASI software. We compared the riboflavin concentration generated under normal enzyme abundance with those optimized for the different enzymes (Fig. 2). Based on the kinetic model, *RibA* is the limiting step in riboflavin production (Fig. 2). Under steady-state conditions, riboflavin concentration reaches 25.13 μmol/L. By overexpressing *PYRD, PYRR*, and *RS*, it reaches 25.30, 26.10, and 26.13 μmol/L, respectively (Fig. 2), which is comparable to the non-optimized model. However, the overexpression of *RibA* leads to higher levels of riboflavin accumulation, at 28.2 μmol/L (Fig. 2). These findings indicate that increasing the flux in *PYRD* and *PYRR* does not result in a higher concentration of riboflavin, as the biosynthesis bottleneck occurs upstream. Furthermore, increasing RS abundance does not lead to a higher riboflavin concentration, likely due to low substrate availability. Overall, these results highlight the importance of *RibA* for riboflavin production and suggest that boosting *RibA* activity could be a promising strategy for enhancing riboflavin accumulation in the system.

### Arabidopsis *RibA1* sequence and expression pattern is mirrored by *OsRibA1* and *OsRibA2*

**Fig. 3:**
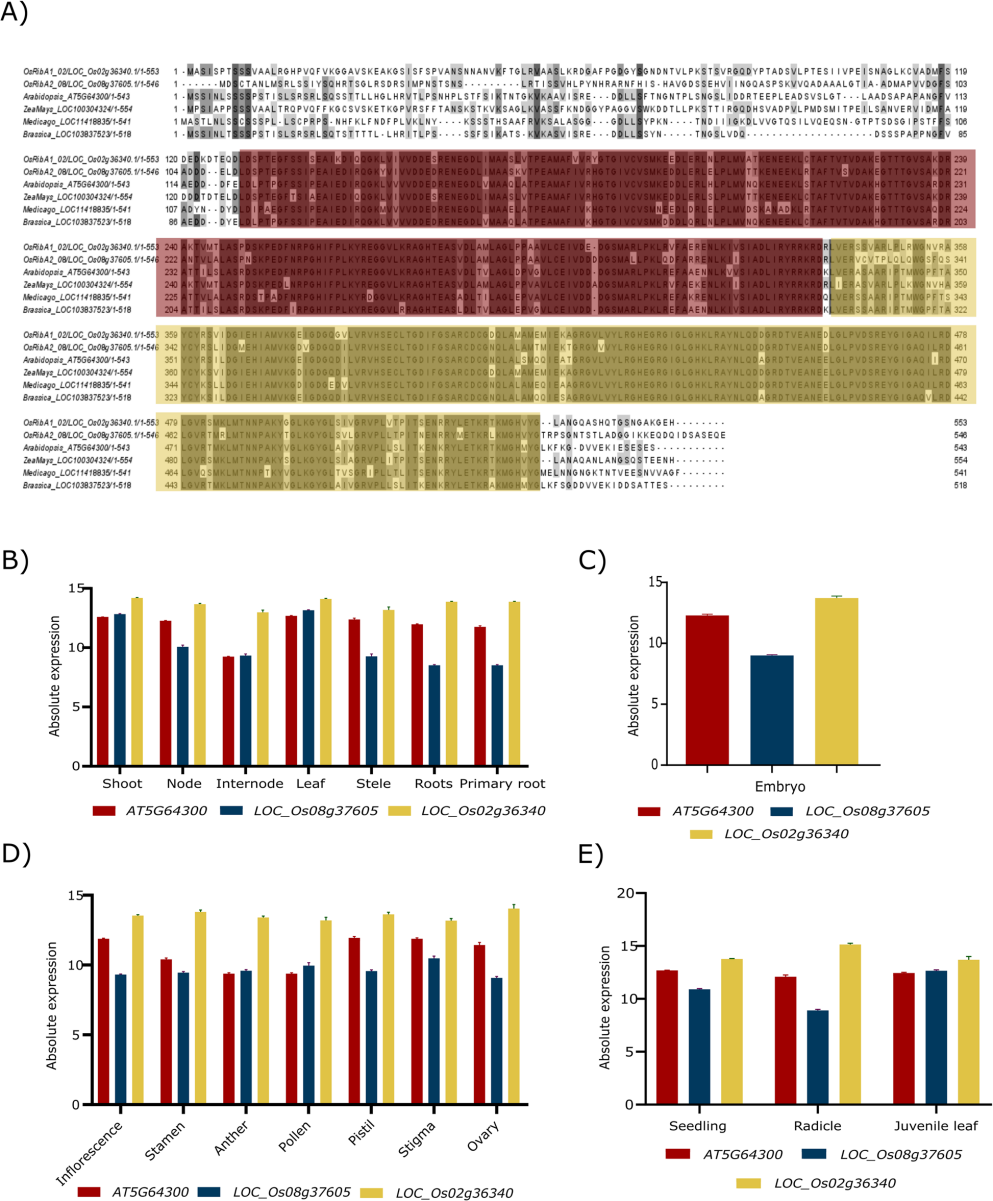
Comparative analysis of RibA protein sequences and expression profiles. **A** Alignment of OsRibA homologs. RibA protein sequences of *Oryza sativa* (LOC_Os02g36340, LOC_Os08g37605) (putative), *Arabidopsis thaliana* (AtRibA1), *Zea mays* (ZmRibA1), *Brassica rapa* (BrRibA1) and *Medicago truncatula* (MtRibA1). The alignment was performed using ClustalW and visualized in JalView. The red boxes are the conserved sequences of DHBS domain and green boxes the conserved sequences of GTPCHII domain. Expression patterns of profile Arabidopsis RibA1 (AT1522940), OsRibA1 (LOC_Os08g37605) and OsRibA2 (LOC_Os02g36340) in **B**) shoot and root, **C** seedling, **D** inflorescence and **E**) seed organs, of Arabidopsis gene RibA1 (AT1522940), OsRibA1 (LOC_Os08g37605) and OsRibA2 (LOC_Os02g36340), according to Genevestigator data

After identifying RibA as the bottleneck of the riboflavin pathway in plants, since *RibA* had not yet been found in rice, we searched for rice orthologs of Arabidopsis *RibA1* (AT5G64300). AtRibA1 is a bifunctional enzyme involved in (1) the hydrolytic release of formate and inorganic pyrophosphate from GTP to form a pyrimidine moiety and in (2) the skeletal rearrangement of ribulose 5-phosphate to form DHB4P (Fig. 1; [35]). Using the BLASTt algorithm, we searched *Oryza sativa* japonica cv. Nipponbare genome and found three potential candidates: LOC_Os08g37605, LOC_Os02g36340 and LOC_Os05g38570. Sequence similarities analysis revealed that LOC_Os08g37605 shares 74.37% identity to *Arabidopsis* AT5G64300 at the genome level and 69.89% at the amino acid level. LOC_Os02g36340 has 84.2% similarity at the genome level and 82.1% at the amino acid level while LOC_Os05g38570 has 70.26% similarity at the genome level and 75.63% at the amino acid level. Sequence analysis revealed that the three candidates are putative bifunctional enzymes with dual domains (Figure S4). However, phylogenetic analysis of the three candidates revealed that LOC_Os05g38570 is probably not bifunctional GTPCHII/DHBPS, as it clusters with the monofunctional RibA3 protein from Arabidopsis and other plant species (Figure S5). Furthermore, similar to Arabidopsis *AtRibA3*, LOC_Os05g38570 has the same one residual change at the ribulose-5-phosphate binding region and one residual alteration at the conserved catalytic site, which is required for the DHBPS activity (Figure S4) [35, 78]. Regarding GTPCHII domain, the three candidate genes do not show the residual alterations in the zinc-binding domain or catalytic sites responsible for the loss of this activity (Figure S4) [35]. Based on these results, LOC_Os08g37605 was named *OsRibA1* and LOC_Os02g36340 *OsRibA2*. *OsRibA1* and *OsRibA2* encode proteins with 546 and 554 amino acids, respectively, and contain 2 conserved domains according to Pfam and ScanProsite, a DHBPS and a GTPCHII domain (Fig. 3A). To investigate the expression patterns of *OsRibA1* and *OsRibA2*, we used Genevestigator, a gene expression database that presents the data in the context of plant development, plant organ and environmental conditions [64]. We compared the expression of *OsRibA1* and *OsRibA2* with the known one from Arabidopsis (*AtRibA1*) (Fig. 3B-E) and verified that both genes have similar expression profiles across various plant organs, including root, shoot, seedling, seed and inflorescence. The similar accumulation of Os*RibA* transcripts across all organs indicates little tissue-specific expression. Our results support the hypothesis that *OsRibA1* and *OsRibA2* both function as GTPCHII and DHBPS in rice.

**Fig. 4:**
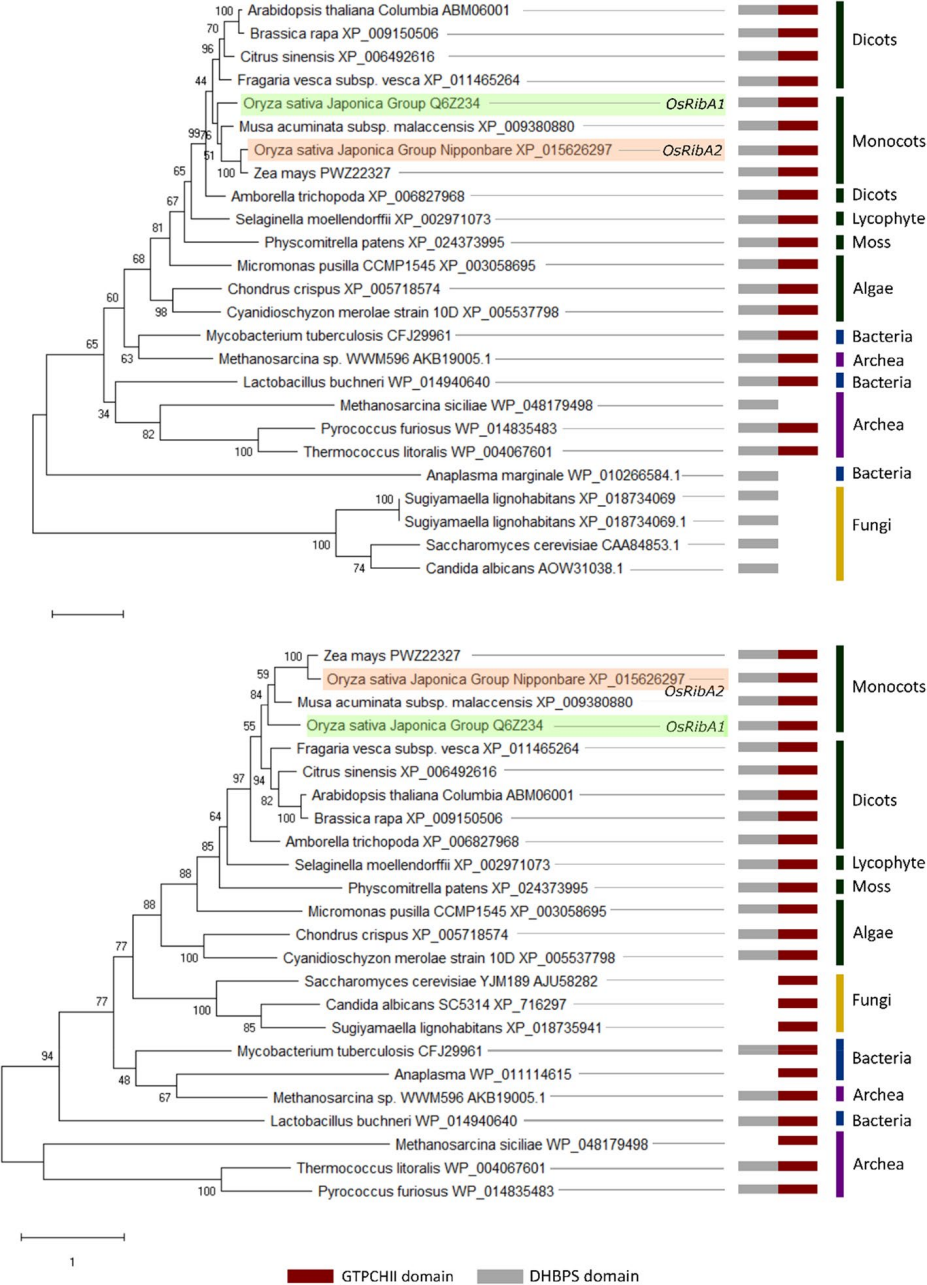
Phylogenetic analysis trees and domain composition of RibA proteins. Maximum likelihood phylogenetic trees developed using MEGAX with the sequences of the bifunctional proteins identified across several kingdoms and **A**) the sequences of GTPCHII domain present in some microorganisms or. **B** Phylogenic tree developed with the sequences of the bifunctional proteins present in several kingdoms and the sequences of DHBPS from microorganisms. The numbers at the branching points indicate the percentage of times that each branch topology was found during bootstrap analysis (*n* = 1000). The boxes represent predicted functional domains: red – GTPCHII domain; grey –DHBPS domain

Aiming to establish ortholog relationships between RibA proteins from other plant species, phylogenetic trees were constructed. This analysis comprised known and putative GTPCHII/DHBPS bifunctional proteins from members of the tree kingdoms, Eubacteria, Archaea and Eukarya (Table S6). Both Eubacteria and plants have bifunctional GTPCHII/DHBPS proteins, while fungi have two distinctive enzymes for each reaction [79] (Fig. 4). For this reason, we built phylogenetic trees with the sequences of the bifunctional *RibA* proteins and the GTPCHII proteins (Fig. 4A) or the DHBPS proteins from yeasts (Fig. 4B).

GTPCHII/DHBPS proteins from land plants formed a distinct group in both phylogenetic trees, showing that the GTPCHII/DHBPS bifunctionality is conserved across algae and plant species (Fig. 4A and B). This was also evident for members of Archaea and bacteria (Fig. 4A and B), where most species have GTPCHII/DHBPS bifunctionality. Interestingly, in fungi, only monofunctional enzymes have been reported. In both phylogenetic trees, OsRibA1 and OsRibA2 cluster with the bifunctional proteins from Poaceae supporting their function as GTPCHII/DHBPS bifunctional proteins.

### Functional studies reveal *OsRibA1* as outperforming *OsRibA2* in rescuing growth impairments of GTPCHII- and DHBPS-deficient yeast mutants

To validate the DHBPS and GTPCHII functions of OsRibA1 and OsRibA2, yeast complementation studies were conducted, taking advantage of the well-established knowledge of the riboflavin pathway in this organism [80–83]. Complementation assays were performed using cell spotting and growth curve analysis, using the riboflavin-deficient yeast mutants *rib1∆* (for GTPCHII) and *rib3∆* (for DHBPS).

**Fig. 5:**
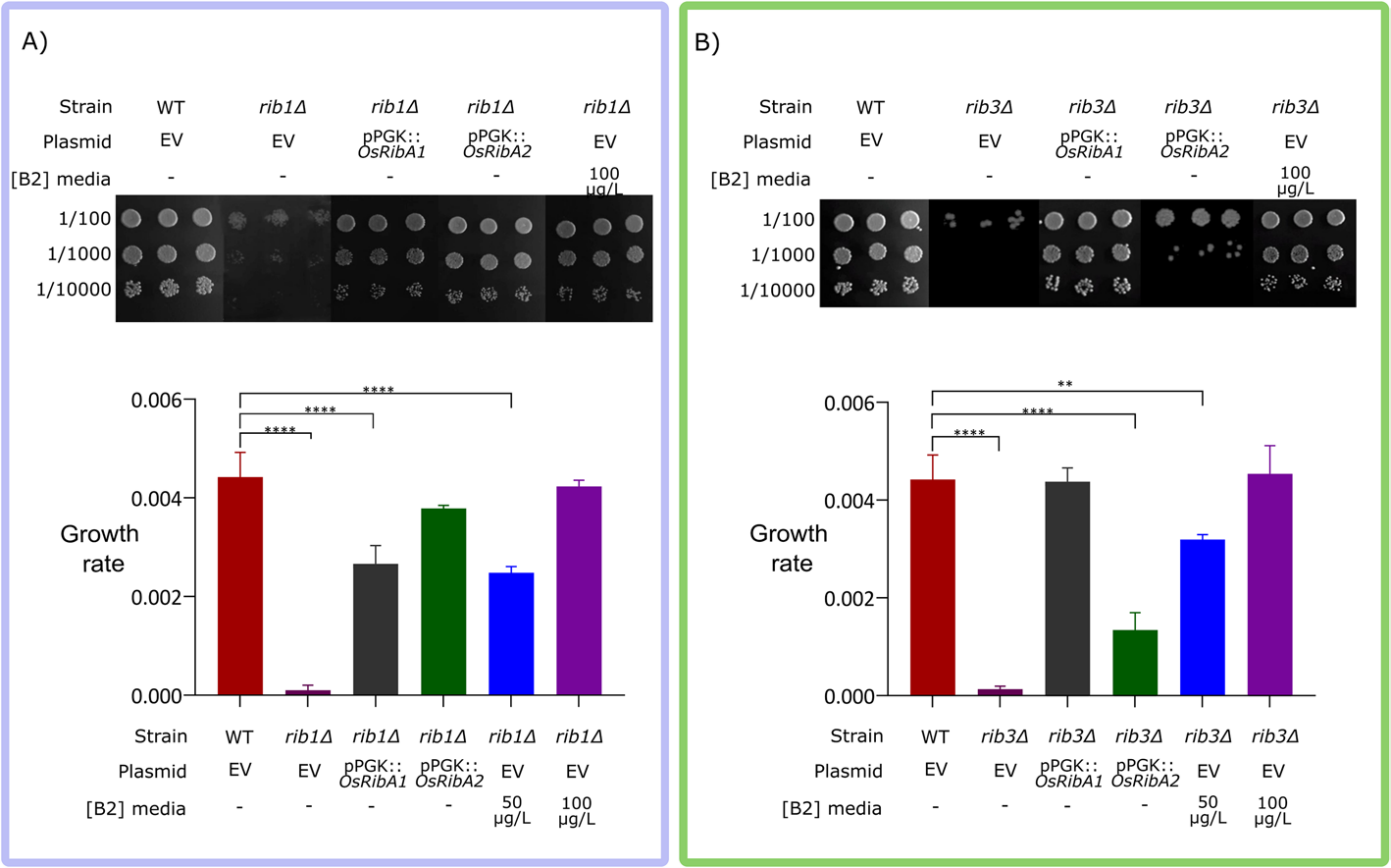
Functional complementation of the yeast mutants *rib1∆* (GTPCHII deficient) and *rib3∆* (DHBPS deficient) with *OsRibA1* and *OsRibA2.* The mutants transformed with the empty vector (EV) were used as negative control with no exogenous supply of riboflavin, while the wild type transformed with the empty vector (EV) and no added thiamin was used as positive control for all experiments. The growth rate was determined by calculating the slope of the non-linear regression curve applied to the exponential phase of the growth curves. **A** Cell spotting assay and growth rate for *rib1∆,* deficient in GTPCHII. **B** Cell spotting and growth rate for *rib3∆,* deficient in DHBPS. Data shows that *RibA2* fully recovers the GTPCHII mutant and that *RibA1* fully restores DHBPS mutant. Data shown correspond to means ± standard deviation of the means (SD) (*N* = 5). Data was compared to control values (WT) by One-way ANOVA (**, *P* < 0.01; ****, *P* < 0.0001)

Regarding *rib1∆* (GTPCHII deficient), the results obtained from the cell spotting assay and growth curve analysis revealed that both *OsRibA1* and *OsRibA2* expression could rescue its growth impairment on riboflavin deficient SD medium (Fig. 5A). Interestingly, *OsRibA2* fully recovered the growth of *rib1∆* while *OsRibA1* only partially restored it (Figs. 5A and S6), indicating that *OsRibA2* and, to a lesser extent, *OsRibA1* have GTPCHII activity. Additionally, the growth of the mutants was also restored by the addition of 100 μg/L of riboflavin to the medium stabilized with 0.05% EDTA [84] (Fig. 5A) due to its high photosensitivity (Figure S7).

In the case of *rib3∆* (DHBPS deficient), the cell spotting assay showed that only *OsRibA1* fully restored the growth of the mutant, whereas *OsRibA2* only slightly improved its growth (Fig. 5B). The results of the growth rate analysis confirmed that *OsRibA1*, and the addition of 100 μg/L of riboflavin to the medium (Figs. 5B and S6), rescued *rib3∆* growth impairment, while*Os RibA2* could not fully complement the growth of the mutant (Figs. 5B and S6). These data indicate *OsRibA1* as the more active bifunctional rice RibA protein, exhibiting superior capability to rescue both GTPCHII and DHBPS activities.

### * OsRibA1* outperforms *OsRibA2* in DHBPS activity while both exhibit comparable GTPCHII functions in vitro

**Fig. 6:**
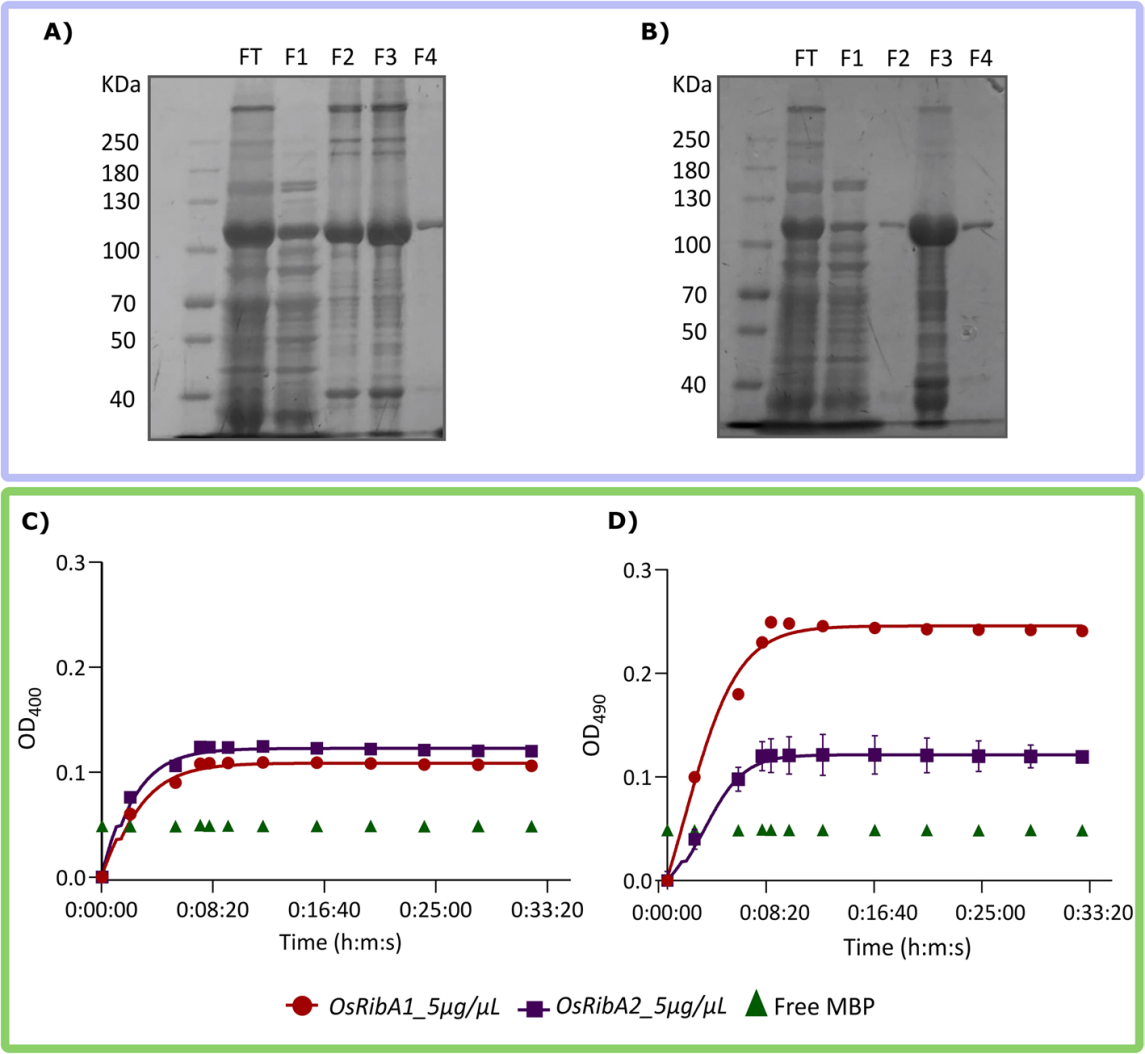
Enzymatic activities of OsRibA proteins. MBP-tagged N-terminal OsRibA proteins were overexpressed in *E. coli* and purified by FPLC. Coomassie staining following SDS-PAGE detects enriched recombinant **A**) OsRibA1 and **B**) OsRibA2 proteins in selected FPLC fractions. FT, Flow through; F1-F4, fraction 1 to 4. The obtained fractions (5μg/μL) were assayed in vitro for **C**) GTPCHII and **D**) DHBPS enzymatic activities. Standard errors are indicated, but only visible in OsRibA2 tested for DHBPS enzyme activity (*N* = 3)

To further validate the ability of OsRibA1 and OsRibA2 to perform both GTPCHII and DHBPS activities, we conducted in vitro activity assays. The enzymes were expressed in *E. coli* and purified by FPLC (OsRibA1 with 101.3 KDa and OsRibA2 with 101.6 KDa) (Figs. 6 and S8). Their GTP cyclohydrolase II (GTPCHII) and 3,4-dihydroxy-2-butanone 4-phosphate synthase (DHBPS) activities were determined in a coupled assay as in Bacher et al*.* [77]. GTPCHII enzymatic function was found to be comparable between OsRibA1 and OsRibA2 (Fig. 6C). However, regarding DHBPS activity, OsRibA1 exhibited higher activity (Fig. 6D). Supporting the yeast complementation results, the in vitro data obtained from these assays confirmed that while both proteins are bifunctional, OsRibA1 displays superior DHBPS activity.

### RibA1 promotes riboflavin accumulation in rice callus

**Fig. 7:**
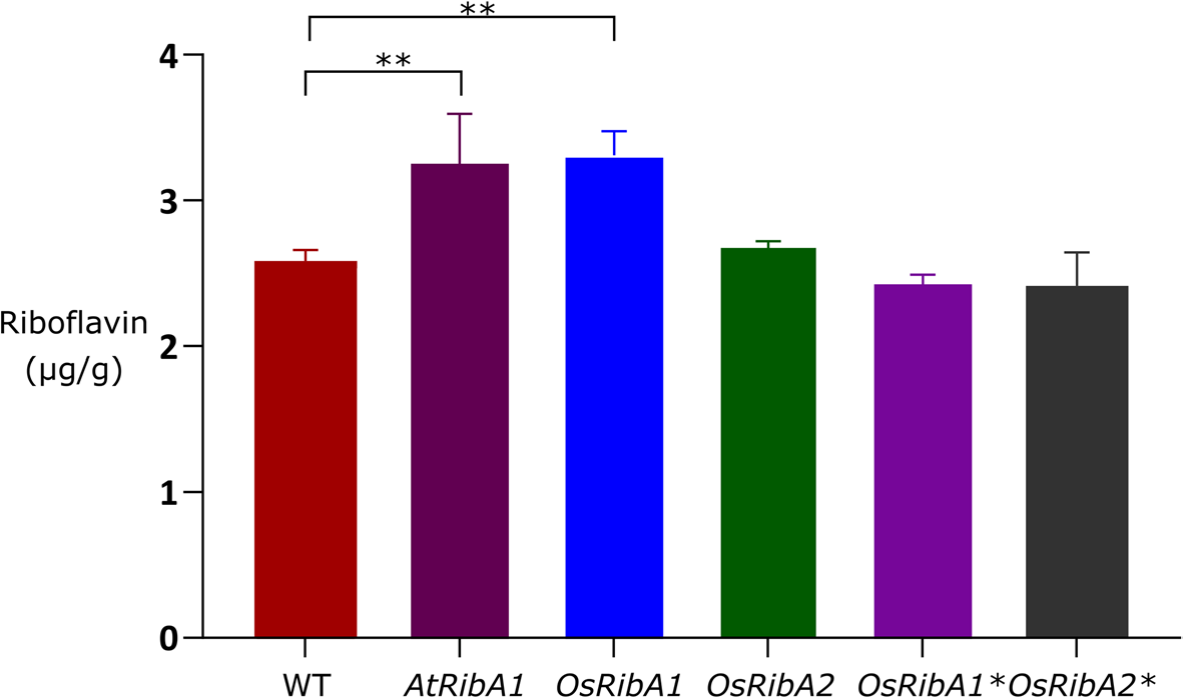
Riboflavin content in rice callus overexpressing *AtRibA1* and *OsRibA1* and *OsRibA2.* As negative controls, *OsRibA1* and *OsRibA2* were also used with a point mutation leading to an early stop codon (indicated as OsRibA1* and OsRibA2*). Twelve independent lines, positive for each transgene, were randomly pooled and stored at -80°C until riboflavin extraction. Riboflavin was detected and quantified by HPLC–MS. Data shown correspond to means ± standard deviation of the means (SD), *N* = 12. Data was compared to control values (WT) by One-way ANOVA (**, *P* < 0.01)

Having previously confirmed the functionality of *OsRibA1* and *OsRibA2* in a heterologous system and in vitro, we sought support of their functionality in rice callus [85]. To the best of our knowledge, our study reports the first observation of increased riboflavin content in rice through the overexpression of Os*RibA1*, reaching a 28% increase as compared to the wild type and negative controls (Fig. 7). The characterized Arabidopsis gene *AtRibA1* (At5g64300) demonstrated similar efficacy in increasing riboflavin content in this tissue upon its overexpression, providing further support for its functional role (Fig. 7). The rice callus overexpressing *OsRibA1* produced 3.31 µg of riboflavin/g dry weight, demonstrating its capacity to enhance riboflavin accumulation, while *OsRibA2* did not affect the vitamin content. Collectively our results reveal *OsRibA1* as a candidate to boost riboflavin levels in plant tissues.

## Discussion

### Kinetic modeling identifies *OsRibA* as the rate-limiting enzyme in riboflavin biosynthesis

Biofortification offers a reliable and cost-effective solution to combat vitamin B2 deficiency. To develop powerful biofortification strategies, it is crucial to gain deeper understanding of B2 metabolism. Metabolic engineering, as a biofortification approach, relies on identifying and stimulating the rate-limiting steps of the target metabolism [31, 86]. Kinetic models, when supplied with appropriate physiological data, enable the mathematical prediction of how different components affect the accumulation of the target metabolite [87], in this case riboflavin. This quantitative description of metabolic phenotypes is achievable by establishing mechanistic relationships between metabolic rates, enzyme levels, and metabolite concentrations [88].

In this study, we applied a kinetic model to B2 biosynthesis to predict the impact of enzyme overexpression on riboflavin concentration. Our findings revealed that OsRibA is the rate-limiting enzyme of the pathway. Overexpression of *OsRibA* genes resulted in higher production of riboflavin over an extended period. Furthermore, our results suggest that increasing the flux in *PYRD* and *PYRR* did not translate into higher riboflavin concentration, in accordance with a biosynthesis bottleneck occurring upstream, at RibA. Additionally, increasing RS abundance did not lead to higher riboflavin concentration, likely due to the limited substrate availability. The utilization of a kinetic model to predict the impact of enzyme overexpression on riboflavin concentration provides a powerful tool for biofortification research [89]. We propose to extend this modeling approach to investigate other metabolic pathways as a support tool for the development of strategies to biofortify diverse crops or organisms for different micronutrients.

While data on the rate-limiting step of riboflavin pathway in plants is currently unavailable, studies on microorganisms, particularly in *Bacillus subtilis*, identified *RibA* as the limiting step [48, 90–92], which is supported by the data obtained with our kinetic model. This consistency across organisms strengthens the reliability of our data and the potential applicability in plant biofortification efforts. Nevertheless, there are several aspects that could be further investigated. To improve the model prediction capability, future research should delve into the specific mechanisms regulating *OsRibA* expression and explore potential cross-interactions.

Recently, biofortification of rice endosperm with riboflavin was performed by using yeast genes [12]. The strategy involved overexpressing the six genes that make up the B2 biosynthetic pathway in yeasts: *ScRIB1*, *ScRIB7*, *ScRIB2*, *ScRIB3*, *ScRIB4*, *ScRIB5* [12]. This approach resulted in a 4.56-fold increase in riboflavin levels in brown rice and 4.24-fold increase in white rice [12]. A strategy based on the sole overexpression of *OsRibA1,* as it represents the bottleneck of the pathway, could eventually be an attractive alternative for cisgenic approaches. Recently, the European Food Safety Authority (ESFA) conducted a hazard comparison between plants produced through cisgenesis, conventional plant breeding techniques, and transgenesis concluding that the hazards of cisgenic and conventionally bred plants are identical [93]. Therefore, and given the increasing public acceptance of cisgenic approaches [43], it would be interesting to develop a strategy requiring a small number of cisgenes to accumulate significant amounts of target micronutrients.

### In silico*, *in vitro and in vivo studies support *OsRibA1* as a key bifunctional player

To characterize *RibA* genes in rice, we performed a comprehensive analysis using yeast complementation assays, in vitro assays, and metabolic engineering screens. The data collected consistently supported the bifunctional capability of *OsRibA1* and *OsRibA2*, and the superior efficacy of *OsRibA1*.

Through BLASTt search using Arabidopsis *RibA1* as query, we identified three putative *RibA* genes in rice cv. Nipponbare genome: *LOC_Os08g37605*, *LOC_Os02g36340* and *LOC_Os05g38570*. We demonstrated that LOC_Os05g38570 (*OsRibA3*) shares the same critical residual changes as Arabidopsis RibA3 at locations required for the DHBPS activity (Figure S3) [35, 78]. These residual changes occur at the ribulose-5-phosphate-binding region and at the conserved catalytic site, both required for DHBPS activities and linked to the loss of function. Intriguingly, while rice has 3 *RibA* homologs like Arabidopsis, Os*RibA1* and Os*RibA2* do not exhibit the characteristic residual changes in the GTPCHII domain associated with loss of function, as seen in *AtRibA2* [35], thus indicating that both *OsRibA1* and *OsRibA2* have maintained their dual functionality.

Phylogenetic analysis revealed that *OsRibA1* and *OsRibA2* are closely related to bifunctional enzymes from other plants, including to *RibA* from maize, which aligns with the close evolutionary relationship between maize and rice [94] (Fig. 4A and B). Interestingly, despite the highly conserved nature of riboflavin biosynthesis, striking evolutionary differences can be found. For instance, *E. coli* and yeasts use two separate enzymes (RibA and RibB), whereas the fused enzyme has been suggested to provide a kinetic advantage by consuming the two substrates stoichiometrically [38]. Additionally, the presence of both *OsRibA1* and *OsRibA2* genes with bifunctional capabilities in the rice genome raises questions about the evolutionary significance and functional diversity within the *RibA* gene family. The fact that both *OsRibA1* and *OsRibA2* are expressed in the same tissues further adds to the puzzle (Fig. 3). Further investigation into the functional specialization of *OsRibA1* and *OsRibA2* and their regulation in different tissues could shed light on the evolutionary forces driving the retention of multiple *RibA* genes in rice and their contribution to riboflavin biosynthesis.

Yeast complementation assays were employed to assess the functionality and enzymatic performance of *OsRibA* genes. By using yeast mutants lacking either GTPCHII (*rib1∆*) or DHBPS (*rib3∆*) functionality, we have tested these activities independently, confirming that both genes have the capacity to perform GTPCHII and DHBPS activities, but with different properties. Although none of the genes fully complemented the growth rate of both mutants, the results indicate that *OsRibA1* has a higher ability to perform both activities, highlighting the distinct functional preferences of OsRibA1 and OsRibA2 and shedding light on the intriguing evolutionary divergence and specialization of *RibA* homologs in rice.

To further confirm the enzymatic activities observed in the yeast complementation assays, we conducted in vitro activity assays using purified recombinant OsRibA proteins. The results from these assays corroborated the findings from the complementation studies. For GTPCHII activity, OsRibA1 and OsRibA2 showed similar activities, however, regarding DHBPS activity, OsRibA1 performed better, thus showing higher bifunctional capability. This is consistent with the stoichiometry of the reactions that require two molecules of ribulose-5-phosphate and only one of GTP [38, 95].

The superior bifunctional potential of *OsRibA1* observed in both yeast complementation and in vitro assays, was further supported by overexpressing the gene in rice callus and confirming riboflavin increased levels. In our study we used embryogenic calluses derived from mature seeds, and transformed them with *OsRibA1* and *OsRibA2* driven by the constitutive maize ubiquitin promoter. Interestingly, only *OsRibA1* was able to improve riboflavin accumulation (by 28%) while *OsRibA2* did not impact riboflavin concentration. The findings from multiple experimental approaches reinforce each other, pinpointing *OsRibA1* as a major candidate for B2 accumulation in rice cells.

## Conclusion

In this work, we developed a kinetic model of the riboflavin biosynthetic pathway and identified *OsRibA* as the rate-limiting step, highlighting this gene as a candidate for riboflavin biofortification. A comprehensive characterization of *OsRibA* genes was conducted revealing that their amino acid sequences are similar to the GTPCHII/DHBPS from Arabidopsis and maize. By use of yeast knockout mutants deficient in either DHBPS or GTPCHII, we have demonstrated that expression of *OsRibA2* fully restores growth in the GTPCHII-deficient mutants, while expressing *OsRibA1* only efficiently rescues growth of the DHBPS-deficient mutants. These findings were further supported by in vitro activity assays, which revealed differences in GTPCHII and DHBPS activities between OsRibA1 and OsRibA2, with OsRibA1 exhibiting higher performance in DHBPS activity. In addition, overexpression of *OsRibA1* in rice callus yielded a 28% increase of in riboflavin content, supporting the aforementioned findings. Taken together, our results provide a comprehensive functional characterization of *OsRibA1* and *OsRibA2* in rice and highlight *OsRibA1* as a candidate for metabolic engineering approaches aiming at enhancing vitamin B2 content.

### Supplementary Information

**Supplementary Material 1.**

**Supplementary Material 2.**

## Data Availability

All data generated or analysed during this study are included in this published article and its supplementary information files.

## Abbreviations

ARP: Amino-3,4-dihydroxy-2-butanone 4-phosphate
ARPP: 5-Amino-6-ribitylamino-2,4(1H,3H)-pyrimidinedione 5’-phosphate
A6RP5P: 5-Amino-6-ribosylamino-2,4(1H, 3H)-pyrimidinedione 5'-phosphate
DA6RP5P: 2,5-Diamin-6-ribosylamino-4(3H)-pyrimidinone 5’-phosphate
DHB4P: Dihydroxy-2-butanone 4-phosphate
DHBPS: 3,4-Dihydroxy-2-butanone 4-phosphate synthase
DMRYL: 6,7-Dimethyl-8-ribityllumazine
FMN: Flavin mononucleotide
FAD: Flavin adenine dinucleotide
GTPCHII: GTP cyclohydrolase II
LS: Lumazine synthase
MBP: Maltose binding protein
*OsRibA*: Rice GTP cyclohydrolase II/3,4-dihydroxy-2-butanone 4-phosphate synthase
Rub5P: Ribulose 5-phosphate
PYRD: 2,5-Diamino-6-hydroxy-4-(5-phosphoribosylamino)pyrimidine deaminase
PYRR: 5-Amino-6-(5-phosphoribosylamino)uracil reductase
RS: Riboflavin synthase
