# Supplementary material for: Mathematical kinetic modelling followed by in vitro and in vivo assays reveal the bifunctional rice GTPCHII/DHBPS enzymes and demonstrate the key roles of OsRibA proteins in the vitamin B2 pathway
Source: BMC Plant Biol. 2024 Mar 26;24:220. doi: 10.1186/s12870-024-04878-z (PMC10964609; doi:10.1186/s12870-024-04878-z)
Supplement: Supplementary file 1 — Supplementary Material 1. [file 12870_2024_4878_MOESM1_ESM.docx]

**Supplementary Information**

**Mathematical kinetic modelling followed by *in vitro* and *in vivo* assays reveal the bifunctional rice GTPCHII/DHBPS enzymes and demonstrate the key roles of OsRibA proteins in the vitamin B2 pathway**

Maria Faustino^1,2^, Tiago Lourenço^1^, Simon Strobbe^2^, Da Cao^2^, André Fonseca^3^, Isabel Rocha^3^, Dominique Van Der Straeten^2*^, M. Margarida Oliveira^1*^

^1^Instituto de Tecnologia Química e Biológica António Xavier, Universidade Nova de Lisboa, 2780-157, Oeiras, Portugal.

^2^Laboratory of Functional Plant Biology, Department of Biology, Ghent University, K. L. Ledeganckstraat 35, B-9000 Gent, Belgium.

^3^ Laboratory of Systems and Synthetic Biology, Instituto de Tecnologia Química e Biológica António Xavier, Universidade Nova de Lisboa, 2780-157, Oeiras, Portugal.

^*^Correspondence:

Dominique Van Der Straeten: [dominique.vanderstraeten@ugent.be](mailto:dominique.vanderstraeten@ugent.be); M. Margarida Oliveira: [mmolive@itqb.unl.pt](mailto:mmolive@itqb.unl.pt)

**Supplementary Figures**

*Constrain-based stoichiometric model*

A stoichiometric model of riboflavin pathway was developed as a preliminary study to identify target genes for biofortification. For the reconstruction of the pathway, the genome information was gathered from MSU Rice Genome Annotation Project and NCBI while the refinement of the draft metabolic network was achieved through public databases, such as KEGG, MetaCyc, Uniprot and BRENDA. The reconstructed metabolic network generated a stoichiometric matrix. With the goal of analyzing flux distributions in the metabolic pathways, we applied Flux balance analysis (FBA) using COBRApy. This method allows the introduction of an optimization problem through linear programming. As the objective function, we set the maximization of RS (riboflavin synthase) flux.

**
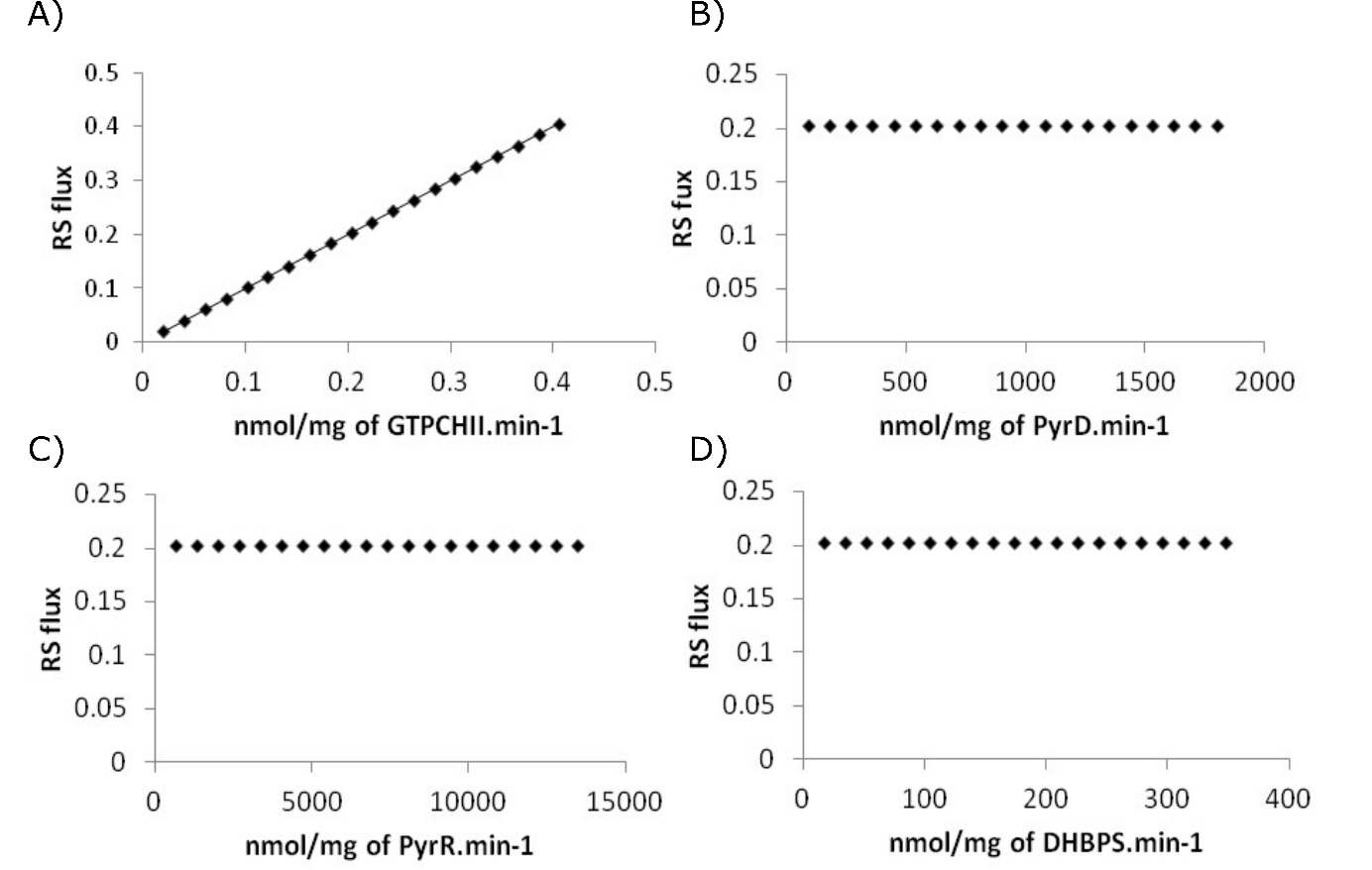
**

**Figure S1. Solution to the flux balance analysis (FBA) optimization problem, maximization of RS flux.** The objective of the model optimization was set as the maximization of riboflavin synthase (RS) flux, that leads to the production of riboflavin. The correlation of each enzyme activity with RS flux was carried out by setting the flux of each reaction as values corresponding to 0%, 20%, 40%, 60%, 80%, 100%, 120%, 140% and 180% of enzyme activity. A combination of all possible enzymatic activities was applied, and FBA was run to simulate the optimal flux in RS. Reactions that had linear correlation with fluctuations in the objective flux were considered rate-limiting**. A)** Correlation between GTPCHII (GTP cyclohydrolase II) activity and RS flux. GTPCHII activity directly correlates with RS flux, pointing it as the limiting step of the pathway. **B)** Correlation between PyrD (2,5-diamino-6-hydroxy-4-(5-phosphoribosylamino)pyrimidine deaminase) activity and RS flux. No correlation was observed. **C)** Correlation between PyrR (5-amino-6-(5-phosphoribosylamino)uracil reductase) activity and RS flux. No correlation was observed. **D)** Correlation between DHBPS (3,4-dihydroxy-2-butanone 4-phosphate synthase) and RS flux. No correlation was observed.

**
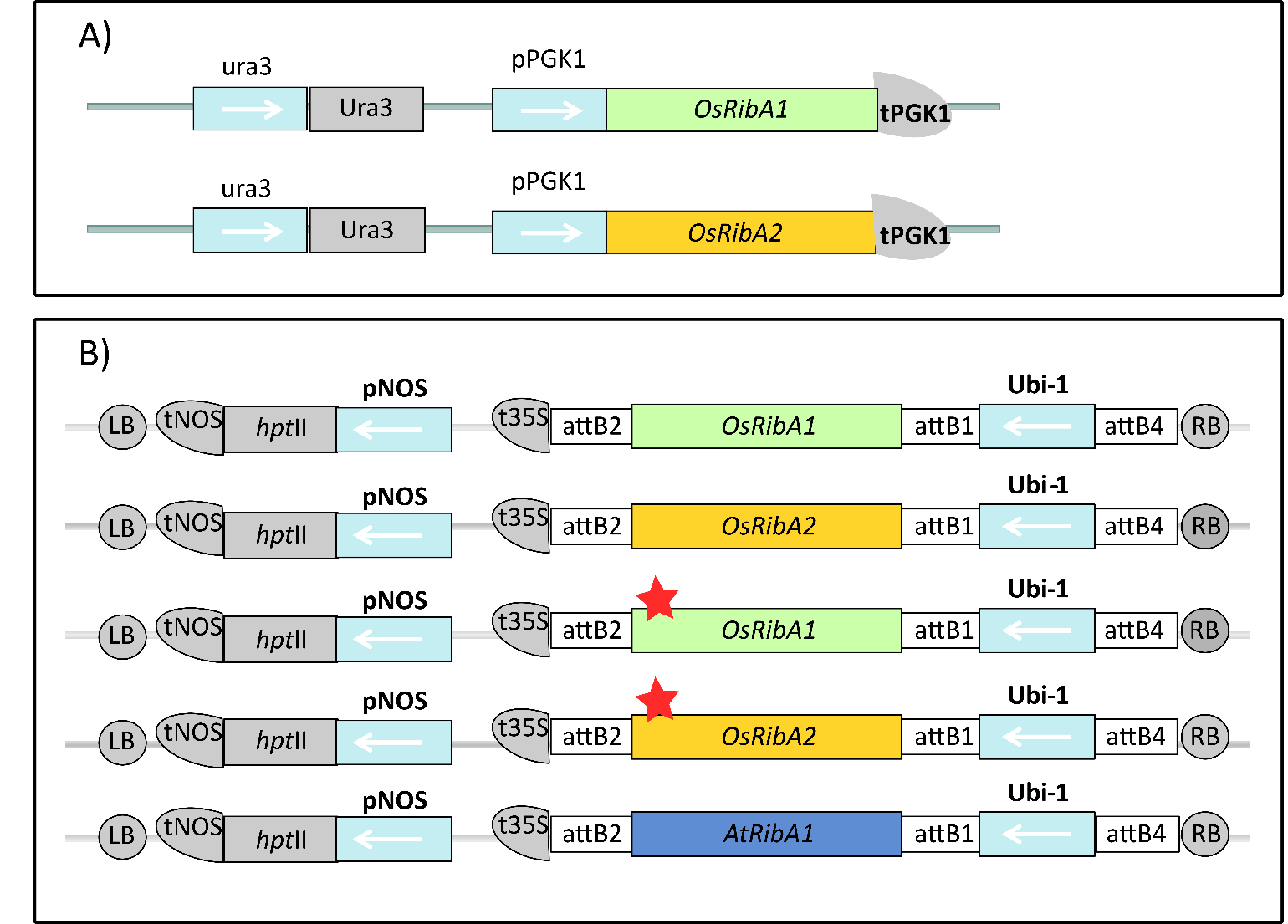
**

**Figure S2. Schematic representation of the constructs used in this study**. **A)** Constructs applied in yeast complementation assays. **B)** Constructs used for overexpression in rice callus. Light blue boxes represent promoters. Colored arrows illustrate coding sequences (CDS) (OsRibA1, green; OsRibA2, orange; AtRibA1, blue). Half-moons depict terminators, grey boxes antibiotic resistance and stars the introduction of a point mutation that leads to the formation of an early stop codon. Abbreviations: LB, left border; RB, right border; attB1, attB2 and attB4 stand for Gateway specific recombination sites; *hpt*II, hygromycin phosphotransferase gene (conferring hygromycin B resistance); Ura3, orotidine-5′-phosphate decarboxylase; t35S, cauliflower mosaic virus 35S terminator; Ubi-1, maize ubiquitin promoter 1; pPGK1, phosphoglycerate kinase promoter; tPGK1, phosphoglycerate kinase terminator; ura3, orotidine-5′-phosphate decarboxylase promoter; pNOS, nopaline synthase promoter; tNOS, nopaline synthase terminator; *OsRibA1*, rice bifunctional GTP cyclohydrolase II/3,4-dihydroxy-2-butanone 4-phosphate synthase (LOC_Os08g37605); *OsRibA2,* rice bifunctional GTP cyclohydrolase II/3,4-dihydroxy-2-butanone 4-phosphate synthase (LOC_Os02g36340); *AtRibA1*, Arabidopsis bifunctional GTP cyclohydrolase II/3,4-dihydroxy-2-butanone 4-phosphate synthase (AT5G64300).

**
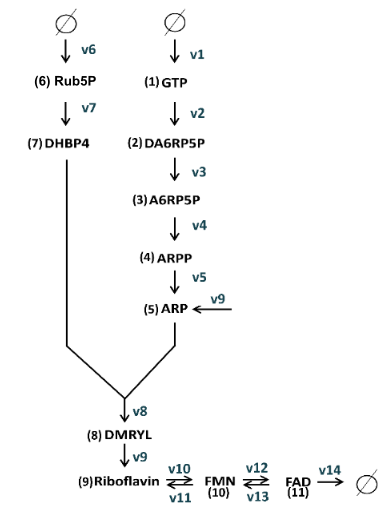
**

**Figure S3. Schematic representation of B2 model**. The kinetic model was constructed based on riboflavin metabolic pathway, and comprises 12 metabolites and 14 reactions, including external transport (reactions v1, v6 and v14). (1) GTP, guanosine-5'-triphosphate; (2) 2,5-diamin-6-ribosylamino-4(3*H*)-pyrimidinone 5’-phosphate (DA6RP5P); (3) 5-amino-6-ribosylamino-2,4(1*H*, 3*H*)-pyrimidinedione 5'-phosphate (A6RP5P); (4) 5-amino-6-ribitylamino-2,4(1H,3H)-pyrimidinedione 5’-phosphate (ARPP); (5) 5-amino-6-ribitylamino-2,4 (1H,3H)-pyrimidinedione (ARP); (6) ribulose 5-phosphate (Rub5P); (7) 3,4-dihydroxy-2-butanone 4-phosphate (DHB4P); (8) 6,7-dimethyl-8-ribityllumazine (DMRYL); (9) riboflavin; (10) flavin mononucleotide (FMN); (12) flavin dinucleotide (FAD). Reaction names: v1, import of GTP; v2, GTP cyclohydrolase II (RibA); v3, PYRD, 2,5-diamino-6-hydroxy-4-(5-phosphoribosylamino)pyrimidine deaminase; v4, PYRR, 5-amino-6-(5-phosphoribosylamino)uracil reductase; v5, PYRP, 5-amino-6-(5-phospho-D-ribitylamino)uracil phosphatase; v6, import of ribulose-5-phosphate; v7, DHBPS (RibA), 3,4-dihydroxy-2-butanone 4-phosphate synthase; v8, LS, lumazine synthase; v9, RS, riboflavin synthase; v10 and v11, RK, riboflavin kinase; v12 and v13, FADS, FAD synthetase. ; v14, external transport of FAD.

**
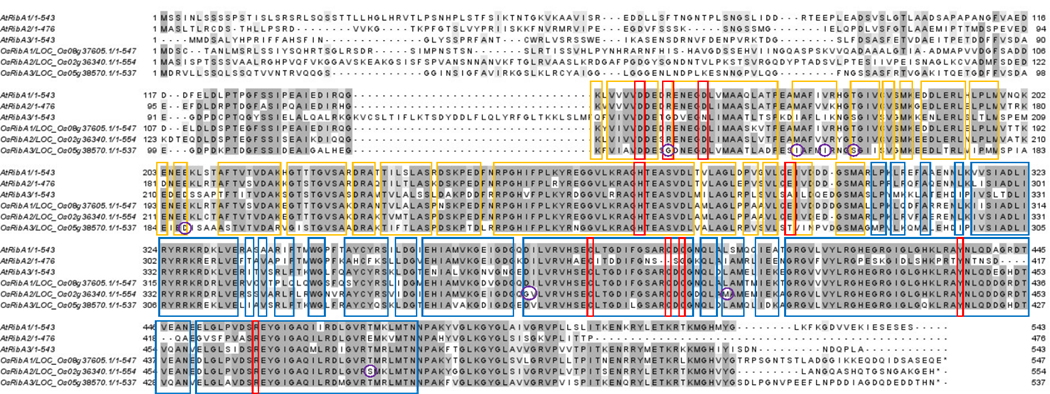
**

**Figure S4. Comparison of the coding domains of rice and Arabidopsis *RibA* genes.** Sequence alignments of DHBPS and GCHII in the three *RibA* proteins from Arabidopsis and rice. The residues essential for enzyme activities are indicated in colored shapes. Blue box, the conserved amino acid sequences for GTPCHII activity; yellow box, the conserved amino acid sequences for DHBPS activity; Red box, enzymatically important amino acid residues; Purple circles, residue substitution or deletion, in a catalytic or substrate-binding domain.

**
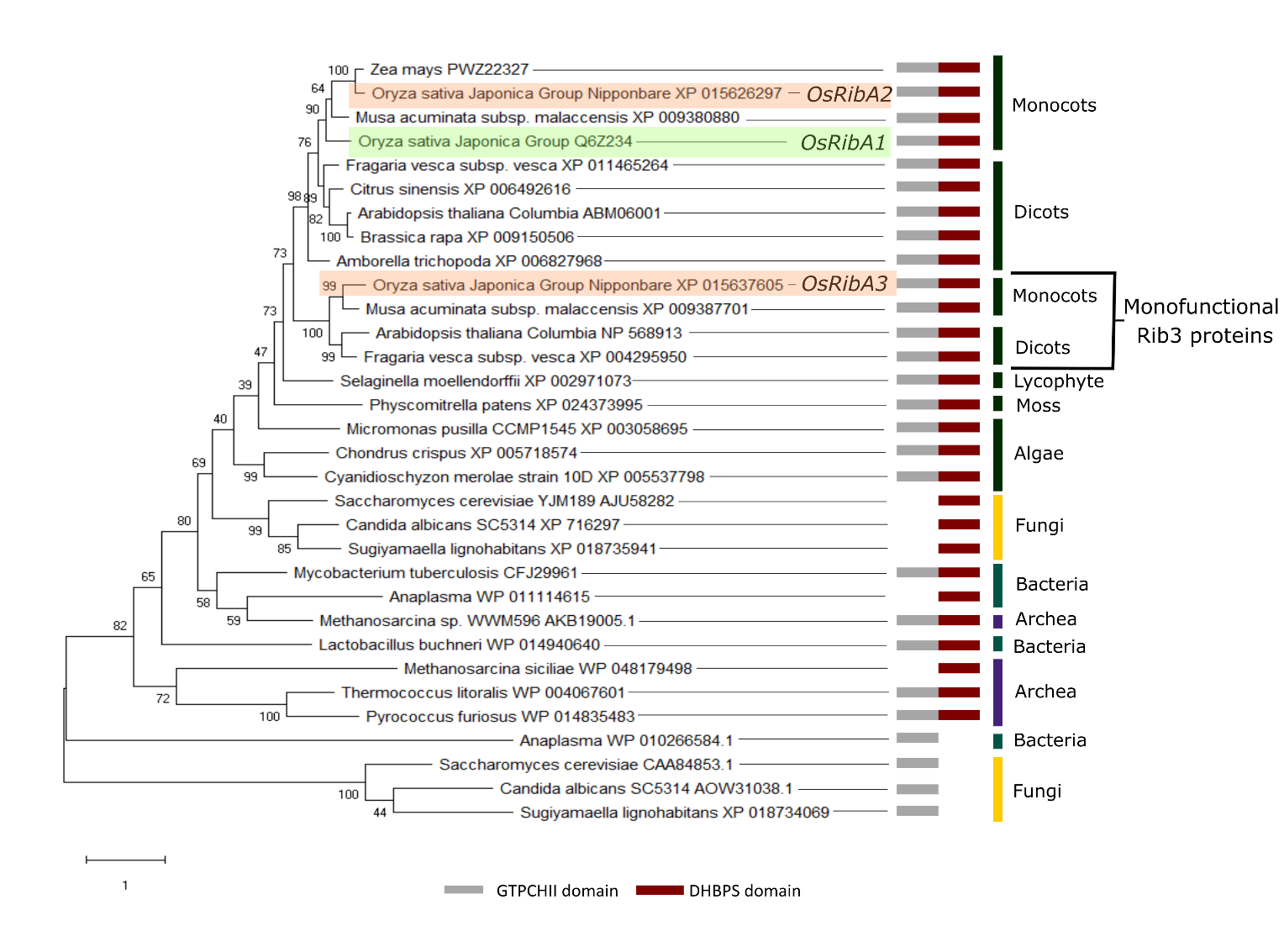
**

**Figure S5**. **Phylogenetic trees and domain composition of RibA proteins.** The numbers at the branching points indicate the percentage of times that each branch topology was found during bootstrap analysis (n=1000). The boxes represent predicted functional domains: red – DHBPS; grey – GTPCHII.


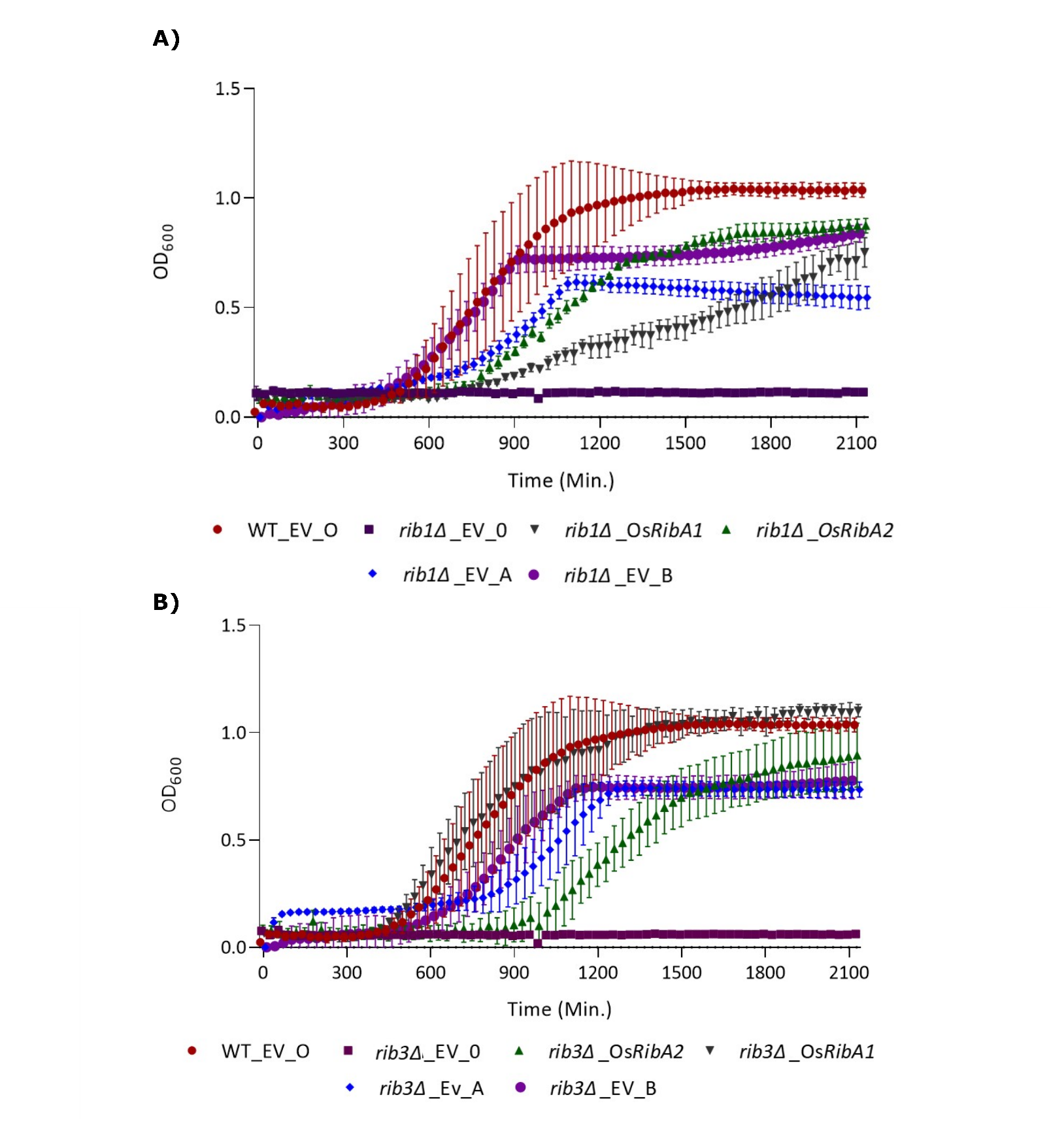


**Figure S6. Functional complementation of** ***rib1∆* (A) and *rib3∆* (B) yeast mutants, growth curves analysis.** The mutants transformed with the empty vector (EV) were used as negative control without exogenous supply of riboflavin (0) (*rib1∆_EV_0* and *rib3∆_EV_0)*, while the wild type transformed with the empty vector (EV) and lacking riboflavin supply (WT_EV_O) was used as positive control for all experiments. In all cases, *rib1∆*_*OsRibA1*_0 and *rib3∆*_*OsRibA1*_0 represent the mutants transformed with *OsRibA1* with no added riboflavin while *rib1∆*_*OsRibA2*_0 and *rib3∆*_*OsRibA2*_0 represent the mutants transformed with *OsRibA2* with no added riboflavin. Rib1*∆*_EV_A, *rib3∆*_EV_A, rib1*∆*_EV_B and *rib3∆*_EV_B represent the complementation of the mutant with addition of 50μg/L riboflavin (_**A**) or 100μg/L riboflavin (**_B**). Growth was monitored by optical density (OD_600nm_) measurements using the TECAN Infinite 200 Pro plate reader. 24-well plates (CELLSTAR, Greiner Bio-One) were inoculated with 500 µL (OD_600nm_ = 0.05) and incubated at 30°C for 36 hours (200 rpm).Standard errors are indicated, N= 5.

**
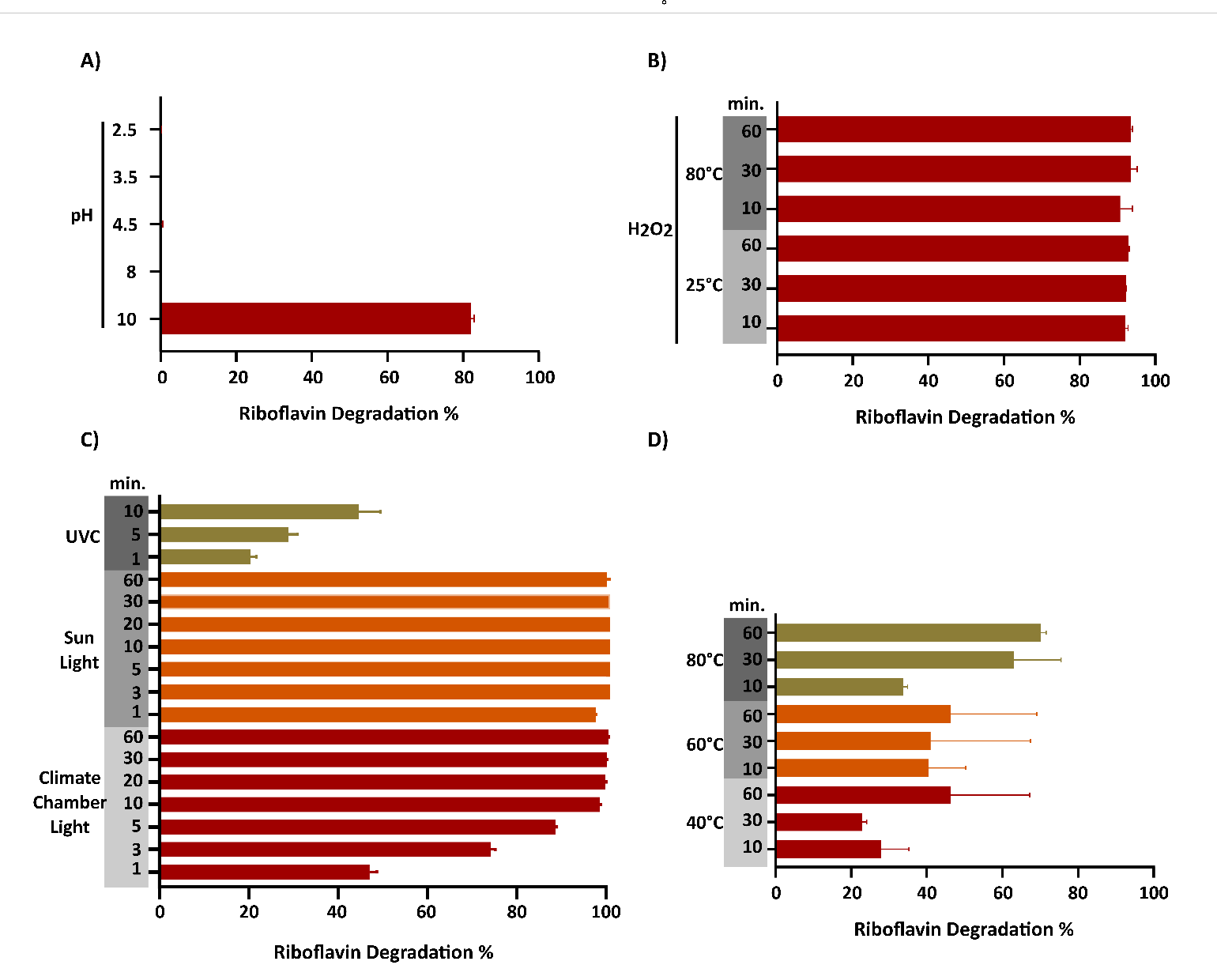
**

**Figure S7. Riboflavin stability assay.** Riboflavin (Sigma-Aldrich, Germany) was dissolved in alkaline solution (0.01N NaOH) to a concentration of 100 mg/mL. Eleven calibration standards (100, 50, 25, 12.5, 6.25, 3.19, 1.56, 0.78, 0.39, 0.19 and 0.097 mg/mL) were prepared by diluting the stock solution in deionized water. Riboflavin solution was subjected to several conditions to evaluate its stability to pH (A), temperature (B), oxidative stress (C) and light (D). A) Riboflavin solution at 100 mg/mL was subjected to changes in pH by separately adding hydrochloric acid and sodium hydroxide to a final pH of 2.5, 3.5, 4.5, 8 and 10. B) Riboflavin solution at 100 mg/mL was exposed to hydrogen peroxide solution (3%) and heated to 25 (T25) or 80 °C (T80) during 10, 30 and 60 min. C) The photodegradation of riboflavin was performed by exposure of 100 mg/L riboflavin samples to sun light, light in a climate chamber and UVC radiation. D) To assess its thermosensitivity, riboflavin solution was heated at 40°C (T40), 60 °C (T60) and 80 °C (T80) for 10, 30 and 60 min. The solutions were directly analyzed without dilution. The analysis was performed using a Waters alliance 2695 system coupled with a Waters 2996 Photodiode Array detector (PDA). The column used was a Nova-Pak C18 Column (3.9 mm X 150 mm) with a particle size of 4 μm and its temperature was maintained at 30 ºC. The mobile phase was composed of (A) ammonium acetate 0.1 M:methanol (5 %) (v/v) and (B) ammonium acetate 0.1 M:methanol (20 %) (v/v). The solvent gradient started with 0 % of solvent B over 2 min, followed by 100 % B over 28 min, 0 % of B over 5 min. The injection volume was 100 μL. UV-vis spectral data were gathered in a range of 250 to 500 nm.

**
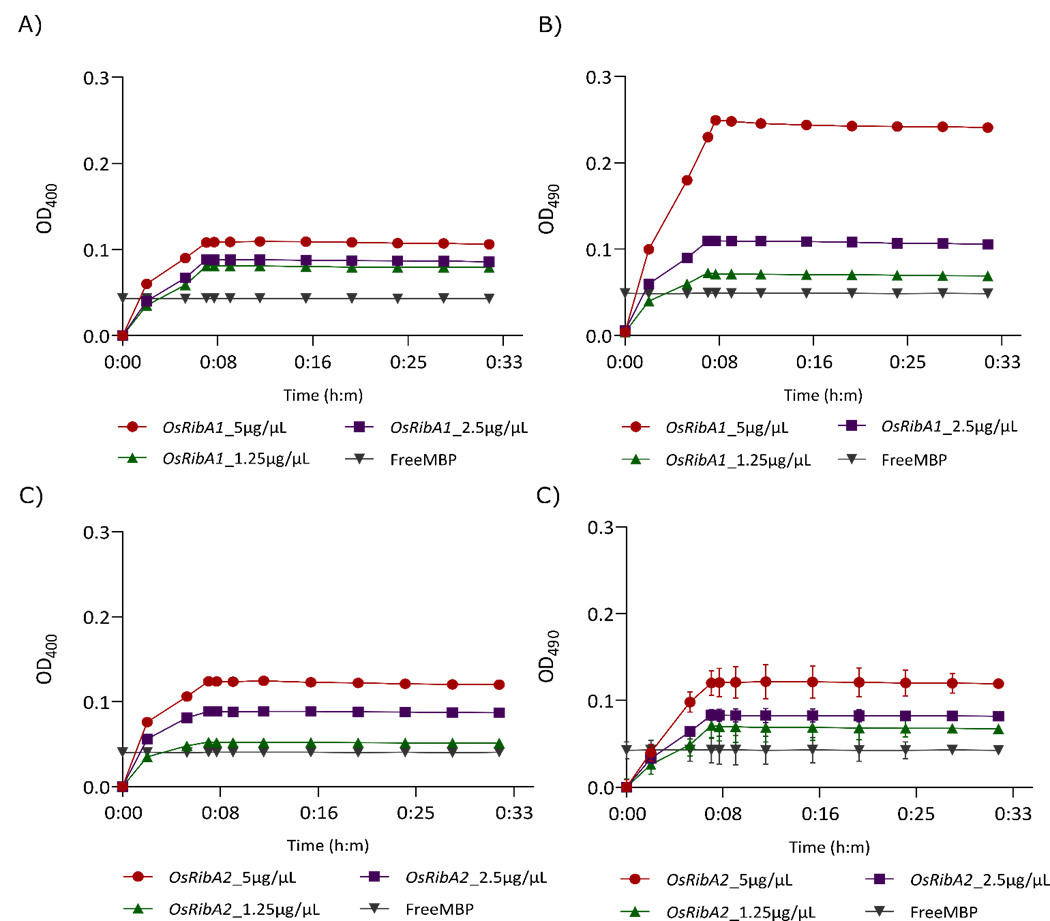
**

**Figure S8. Enzymatic activities of OsRibA proteins.** MBP-tagged N-terminal OsRibA proteins were overexpressed in *E. coli* and purified by FPLC. (A) GTPCHII activity of OsRibA1 at 5, 2.5 and 1.25 μg/μL, (B) DHBPS activity of OsRibA1 at 5, 2.5 and 1.25 μg/μL, (C) GTPCHII activity of OsRibA2 at 5, 2.5 and 1.25 μg/μL ,(D) DHBPS activity of OsRibA2 at 5, 2.5 and 1.25 μg/μL. Standard errors are indicated, N=4.

**Supplementary Tables**

**Table S1.** List of reactions included in the kinetic model.

**Import of GTP:** GTP_in → GTP **(1)**

**GTPCHII(RibA):** GTP + 3H_2_O → 2,5-diamino-6-hydroxy-4-(5-phospho-D-ribosylamino)pyrimidine + formate + P_ii_ **(2)**

**PYRD:** 2,5-diamino-6-hydroxy-4-(5-phospho-D-ribosylamino)pyrimidine + H_2_O → 5-amino-6-(5-phospho-D-ribosylamino)uracil **(3)**

**PYRR:** 5-amino-6-(5-phospho-D-ribosylamino)uracil + NADP^+^ → 5-amino-6-(5-phospho-D-ribitylamino)uracil NADPH + H^+^ **(4)**

**PYRP:** 5-amino-6-(5-phospho-D-ribitylamino)uracil + H_2_O → 5-amino-6-(D-ribitylamino)uracil + P_i_ **(5)**

**Import of Ribulose-5-phosphate:** R5P_in → Ribulose-5-phosphate (**6)**

**DHBPS (RibA):** Ribulose-5-phosphate → (2S)-2-hydroxy-3-oxobutyl phosphate + formate (**7)**

**LS:** 5-amino-6-(D-ribitylamino)uracil + 2S)-2-hydroxy-3-oxobutyl phosphate → 6,7-dimethyl-8-(1-D-ribityl)lumazine + 2H_2_O + P_i_ **(8)**

**RS:** 6,7-dimethyl-8-(1-D-ribityl)lumazine → Riboflavin **(9)**

**RK:** Riboflavin + ATP → Flavin mononucleotide + ADP **(10)**

**FMNH:** Flavin mononucleotide + H_2_O → Riboflavin + P_i_ **(11)**

**FADS:** Flavin mononucleotide + ATP → Flavin dinucleotide + ADP **(12)**

**FADP:** Flavin dinucleotide + H_2_O → Flavin mononucleotide + AMP **(13)**

**Export of FAD:** Flavin dinucleotide → **(14)**

**Table S2.** Rate law (RL) equations, kinetic parameters, and respective references for each reaction of the riboflavin pathway included in the kinetic model.

| Reaction | E. C. Number | Equation | Parameters |
| --- | --- | --- | --- |
| Import of GTP | ?? | Flux1 | Flux= 1.9 |
| GTPCHII (RibA) | 3.5.4.25 | $\frac{V_{max}\times A}{K_{m}+A}$ | V_max_ = 9000  K_m_ = 10000 |
| PYRD | 3.5.4.26 | $\frac{V_{max}\times A}{K_{m}+A}$ | V_max_ = 900  K_m_ = 12800 |
| PYRR | 1.1.1.193 | $\frac{V_{max}\times A}{K_{m}+A}$ | V_max_ =400  K_m_ =5000 |
| PYRP | 3.1.3.104 | $\frac{V_{max}\times A}{K_{m}+A}$ | V_max_ =128000  K_m_ =6730 |
| Import of R5P | ???? | Flux 1 | Flux= 1.9 |
| DHBPS (RibA) | 4.1.99.12 | $\frac{V_{max}\times A}{K_{m}+A}$ | V_max_ =270  K_m_ =250000 |
| LS | 2.5.1.78 | $\frac{V_{max}\times A \times B}{K_{mA} \times K_{mB}+A \times K_{mB}+B \times K_{mA}+A \times B}$ | V_max_ =278  K_mA_ =20000  K_mB_ =26000 |
| RS | 2.5.1.9 | $\frac{V_{max}\times A}{K_{m}+A}$ | V_max_ =378  K_m_ =128000 |
| RK | 2.7.1.26 | $\frac{V_{max}\times A \times B}{K_{mA} \times K_{mB}+A \times K_{mB}+B \times K_{mA}+A \times B}$ | V_max_ =816  K_mA_ =2000  K_mB_ =1030 |
| FMNH | 3.1.3.102 | $\frac{V_{max}\times A}{K_{m}+A}$ | V_max_ =1420  K_m_ =14200 |
| FADS | 2.7.7.2 | Flux | 1.9 |
| FADP | 3.6.1.18 | $\frac{V_{max}\times A}{K_{m}+A}$ | V_max_ =196  K_m_ =9100 |
| Export of FAD | ???? | Flux1 | 1.9 |

**Table S3.** Set of reactions included in the reconstructed pathway of the stoichiometric model.

| Nr | Reaction | EC | co | Equation |
| --- | --- | --- | --- | --- |
| R1 | Transport of GTP into the plastid | - | p | $\to GTP\_p$ |
| R2 | GTPCHII - GTP cyclohydrolase II (RibA) | 3.5.4.25 | p | $GTP\_p\to DA6RP5P\_p$ |
| R3 | PyrD - Diaminohydroxyphosphoribosylaminopyrimidine deaminase | 3.5.4.26 | p | $DA6RP5P\_p\to A6RP5P\_p$ |
| R4 | PyrR - 5-amino-6-(5-phosphoribosylamino)uracil redutase | 1.1.1.193 | p | $A6RP5P\_p\to ARPP\_p$ |
| R5 | R5P transport into plastid | - | p | $\to R5P\_p$ |
| R6 | DHBPS - 3,4-dihydroxy-2-butanone 4-phosphate synthase (RibA) | 4.1.99.12 | p | $R5{P\_}_{p}\to DHBP4\_p$ |
| R7 | Unknown protein | - | p | $ARPP\_p\to ARP\_p$ |
| R8 | LS - Lumazine synthase | 2.5.1.78 | p | $ARP\_p+DHBP4\_p \to DMRYL\_p$ |
| R9 | RS - Riboflavin synthase | 2.5.1.9 | p | $2DMRYL\_p\to Ribolfavin\_p$ |
| R10 | RK - Riboflavin kinase | 2.7.1.26 | p | $Riboflavin\_p \to FMN\_p$ |
| R11 | FMNAT - FAD synthetase | 2.7.7.2 | P | $FMN\_p\to FAD\_p$ |
| R12 | FAD transport from plastid to cytoplasm | - | p | $FAD\_p \to$ |

**Table S4.** Stoichiometric matrix used in the development of the stoichiometric model.

|  | R1 | R2 | R3 | R4 | R5 | R6 | R7 | R8 | R9 | R10 | R11 | R12 |
| --- | --- | --- | --- | --- | --- | --- | --- | --- | --- | --- | --- | --- |
| GTP_p | +1 | -1 | 0 | 0 | 0 | 0 | 0 | 0 | 0 | 0 | 0 | 0 |
| DA6RP5P_p | 0 | +1 | -1 | 0 | 0 | 0 | 0 | 0 | 0 | 0 | 0 | 0 |
| A6RP5P_p | 0 | 0 | +1 | -1 | 0 | 0 | 0 | 0 | 0 | 0 | 0 | 0 |
| ARPP_p | 0 | 0 | 0 | +1 | 0 | 0 | -1 | 0 | 0 | 0 | 0 | 0 |
| ARP_p | 0 | 0 | 0 | 0 | 0 | 0 | +1 | -1 | 0 | 0 | 0 | 0 |
| DMRYL_p | 0 | 0 | 0 | 0 | 0 | 0 | 0 | +1 | -2 | 0 | 0 | 0 |
| Rub5P_p | 0 | 0 | 0 | 0 | +1 | -1 | 0 | 0 | 0 | 0 | 0 | 0 |
| DHB4P_p | 0 | 0 | 0 | 0 | 0 | +1 | 0 | -1 | 0 | 0 | 0 | 0 |
| Riboflavin_p | 0 | 0 | 0 | 0 | 0 | 0 | 0 | 0 | +1 | -1 | 0 | 0 |
| FMN_p | 0 | 0 | 0 | 0 | 0 | 0 | 0 | 0 | 0 | +1 | -1 | 0 |
| FAD_p | 0 | 0 | 0 | 0 | 0 | 0 | 0 | 0 | 0 | 0 | +1 | -1 |

Abbreviations: **GTP_p**: Guanosine-5'-triphosphate; **DA6RP5P_p**: 2,5-diamino-6-hydroxy-4-(5-phospho-D-ribosylamino)pyrimidine; **A6RP5P_p**: 5-amino-6-(5-phospho-D-ribitylamino)uracil; **ARPP_p**: 5-amino-6-(5-phospho-D-ribosylamino)uracil; **ARP_p**: 5-amino-6-(D-ribitylamino)uracil; **DMRYL_p**: 6,7-dimethyl-8-(1-D-ribityl)lumazine; **Rub5P_p**: D-ribulose 5-phosphate; **DHB4P_p**: L-3,4-dihydroxybutan-2-one 4-phosphate; **FMN_p:** Flavin mononucleotide; **FAD_p:** Flavin dinucleotide **R1**: Transport of GTP into the plastid; **R2:** GTPCHII - GTP cyclohydrolase II; **R3:** PyrR - 5-amino-6-(5-phosphoribosylamino)uracil redutase; **R4:** PyrR - 5-amino-6-(5-phosphoribosylamino)uracil redutase; **R5:** R5P transport into plastid; **R6:** DHBPS - 3,4-dihydroxy-2-butanone 4-phosphate synthase; **R7:** Unknown protein; **R8:** LS **-** Lumazine synthase; **R9:** RS - Riboflavin synthase; **R10:** RK - Riboflavin kinase; **R11:** FMNAT - FAD synthetase; **R12:** FAD transport from plastid to cytoplasm.

**Table S5.** List of primers used in this study.

| **Pair nr** | **Primer name** | **Oligonucleotide sequence (5’🡪3’)** |
| --- | --- | --- |
| 1 | LOC_Os02g36340_Fw | ATGGCGTCGATTTCGCCG |
|  | LOC_Os02g36340_Rv | CTAATGCTCCCCTTTGGCAC |
| 2 | LOC_Os08g37605_Fw | ATGGATTCCTGTACTGCAAA |
|  | LOC_Os08g37605_Rv | TTACTCTTGCTCGCTAGC |
| 3 | LOC_Os02g36340_Gib_Fw | ACAACAAATATAAAACACCCATGGCGTCGATTTCGCCG |
|  | LOC_Os02g36340_Gib_Rv | TCAATTCAATTCAATGGATCCTAATGCTCCCCTTTGGCAC |
| 4 | pPGK_LOC_Os02g36340_Gib_Fw | GATCCATTGAATTGAATTGAAATC |
|  | pPGK_LOC_Os02g36340_Gib_Rv | GGGTGTTTTATATTTGTTGTAAAAAG |
| 5 | LOC_Os08g37605_Gib_Fw | GCAAGAGTAAGATCCATTGAATTGAATTGAAATCG |
|  | LOC_Os08g37605_Gib_Rv | AGGAATCCATGGGTGTTTTATATTTGTTGTAAAAAGTAG |
| 6 | pPGK_ LOC_Os08g37605_Gib_Fw | GCAAGAGTAAGATCCATTGAATTGAATTGAAATCG |
|  | pPGK_ LOC_Os08g37605_Gib_Rv | AGGAATCCATGGGTGTTTTATATTTGTTGTAAAAAGTAG |
| 7 | LOC_Os02g36340_GT_Fw | GGGGACAAGTTTGTACAAAAAAGCAGGCTTAATGGCGTCGATTTCGCCG |
|  | LOC_Os02g36340_GT_Rv | GGGGACCACTTTGTACAAGAAGCTGGGTACTAATGCTCCCCTTTGGC |
| 8 | LOC_Os08g37605_GT_Fw | GGGGACAAGTTTGTACAAAAAAGCAGGCTTAATGGATTCCTGTACTGCAAA |
|  | LOC_Os08g37605_GT_Rv | GGGGACCACTTTGTACAAGAAGCTGGGTATTACTCTTGCTCGCTAGCA |
| 9 | AT5G64300_GT_Fw | GGGGACAAGTTTGTACAAAAAAGCAGGCTTAATGTCTTCCATCAATTTATC |
|  | AT5G64300_GT_Fw | GGGGACCACTTTGTACAAGAAGCTGGGTATTAGGACTCAGATTCAGACT |
| 10 | LOC_Os02g36340_PM_Fw | AATTGATCCCTTTGCTTACTTGGAGACTGCACCGC |
|  | LOC_Os02g36340_PM_Rv | GCGGTGCAGTCTCCAAGTAAGCAAAGGGATCAATT |
| 11 | LOC_Os08g37605_PM_Fw | CTGGCCCGATGGTTCTACGGCAAGTGAACAGA |
|  | LOC_Os08g37605_PM_Rv | TCTGTTCACTTGCCGTAGAACCATCGGGCCAG |
| 12 | HPTII_Fw | AATAGCTGCGCCGATGGTTTCTACA |
|  | HPTII_Rv | AACATCGCCTCGCTCCAGTCAATG |

**Table S6.** Species included in the phylogenetic analysis

| Eubacteria | *Anaplasma marginale* |
| --- | --- |
|  | *Lactobacillus buchneri* |
|  | *Mycobacterium tuberculosis* |
| Archaea | *Methanosarcina siciliae* |
|  | *Pyrococcus furiosus* |
|  | *Thermococcus litoralis* |
| Eukarya | *Amborella trichopoda* |
|  | *Arabidopsis thaliana* |
|  | *Candida albicans* |
|  | *Brassica rapa* |
|  | *Chondrus crispus* |
|  | *Citrus sinensis* |
|  | *Cyanidioschyson meroae* |
|  | *Fragaria vesca* |
|  | *Micromonas pusilla* |
|  | *Musa acuminata* |
|  | *Physcomitrella patens* |
|  | *Saccharomyces cerevisiae* |
|  | *Selaginella moellendorffii* |
|  | *Sugiyamaella lignohabitans* |
|  | *Zea mays* |
