# Supplementary material for: Mathematical kinetic modelling followed by in vitro and in vivo assays reveal the bifunctional rice GTPCHII/DHBPS enzymes and demonstrate the key roles of OsRibA proteins in the vitamin B2 pathway
Source: BMC Plant Biol. 2024 Mar 26;24:220. doi: 10.1186/s12870-024-04878-z (PMC10964609; doi:10.1186/s12870-024-04878-z)
Supplement: Supplementary file 2 — Supplementary Material 2. [file 12870_2024_4878_MOESM2_ESM.pdf]

A)

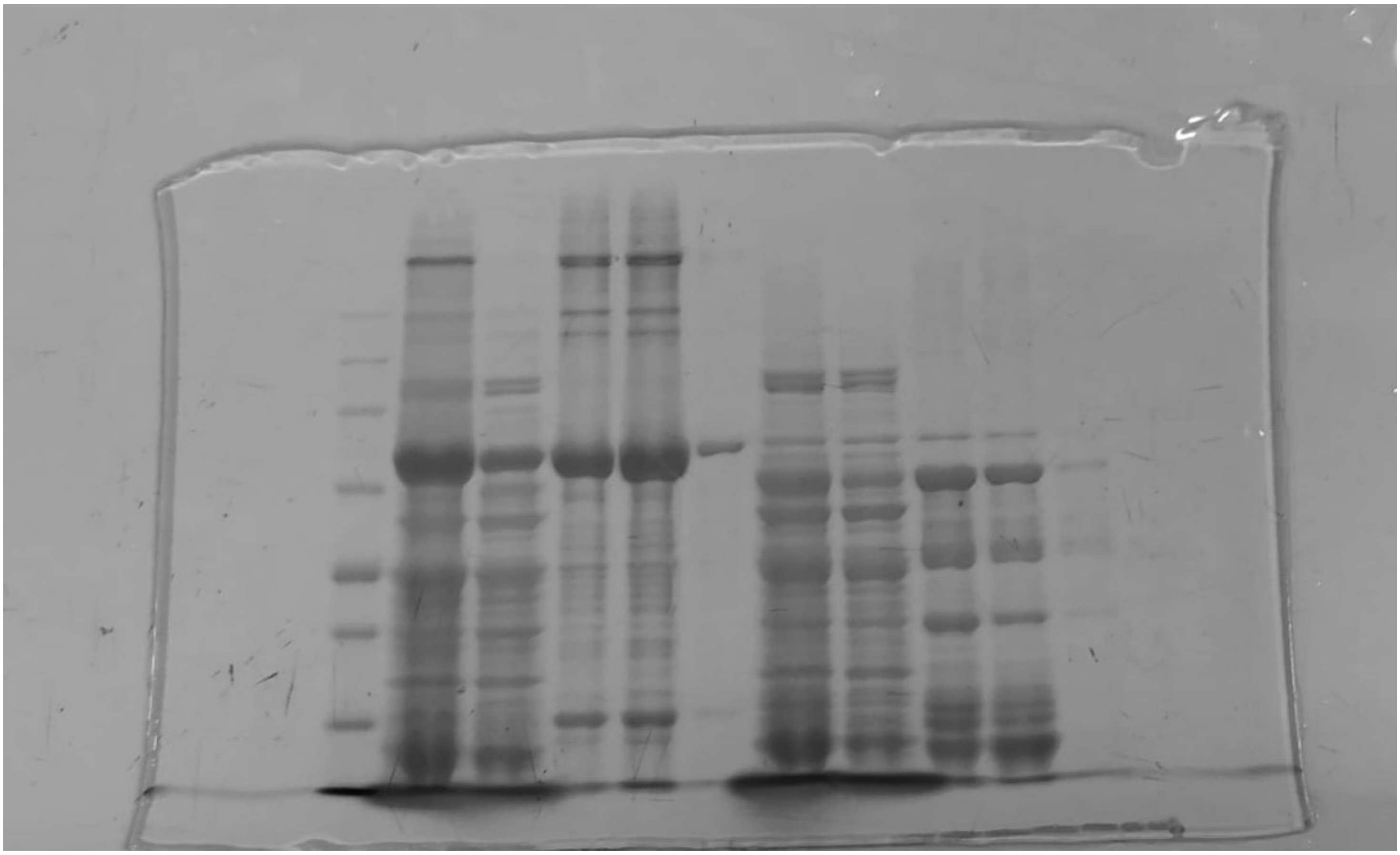

B)

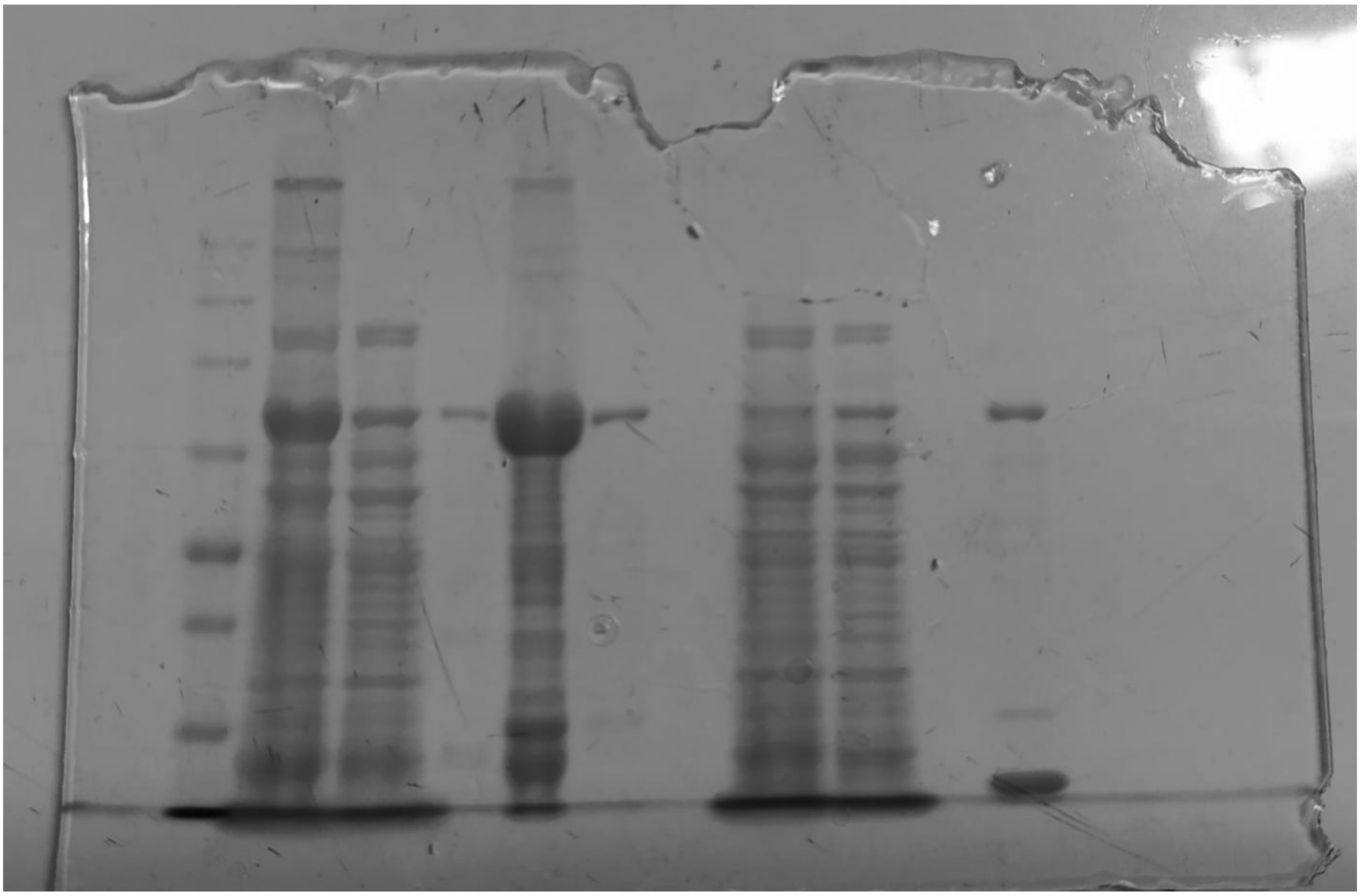

Uncroped gel images of OsRibA1 (A) and OsRibA2 (B) proteins. MBP-tagged N-terminal OsRibA proteins were overexpressed in E. coli and purified by FPLC. Coomassie staining following SDS-PAGE detects enriched recombinant **A)** OsRibA1 and **B)** OsRibA2 proteins in selected FPLC fractions. **A)** OsRibA1 purification.1st column - protein marker; 2nd column - Flow through; 3rd to 6th columns - FPLC fractions; 7th column on - unrelated protein purification. **B)** OsRibA2 purification.1st column - protein marker; 2nd column - Flow through; 3rd to 6th columns - FPLC fractions; 7th column on - unrelated protein purification.
